# The 2025 European Cystic Fibrosis Society position statement on physical activity assessment in cystic fibrosis

**DOI:** 10.1183/16000617.0279-2024

**Published:** 2025-07-09

**Authors:** Craig A. Williams, Brenda Button, Tiffany J. Dwyer, Elpis Hatziagorou, Kelly A. Mackintosh, Melitta A. McNarry, Dewi Paris, James Shelley, Helge Hebestreit, Judy Bradley, Mayara Silveira Bianchim

**Affiliations:** 1 Children's Health and Exercise Research Centre, Faculty of Health & Life Sciences, University of Exeter, Exeter, UK; 2 Department of Respiratory Medicine, Alfred Health and Department Of Medicine, Nursing and Health Sciences, Monash University, Melbourne, Australia; 3 Discipline of Physiotherapy, School of Health Sciences, Faculty of Medicine and Health, University of Sydney, Sydney, Australia; 4 Department of Respiratory Medicine, Royal Prince Alfred Hospital, Sydney, Australia; 5 Paediatric Pulmonology and CF Unit, Hippokration General Hospital of Thessaloniki, Aristotle University of Thessaloniki, Thessaloniki, Greece; 6 Applied Sports, Technology, Exercise and Medicine (A-STEM) Research Centre, Faculty of Science and Engineering, Swansea University, Swansea, UK; 7 School of Health and Sport Sciences, Liverpool Hope University, Liverpool, UK; 8 Department of Pediatrics and Center for Rare Diseases, University Hospital, Würzburg, Germany; 9 The Wellcome Trust-Wolfson Northern Ireland Clinical Research Facility, Belfast City Hospital, and Queen University Belfast, Belfast, UK; 10 School of Health Sciences, Bangor University, Bangor, UK

## Abstract

**Background:**

Recent advances in the measurement of physical activity have significantly enhanced the analyses and interpretation in relation to health and well-being. Thus, we sought to revise and expand the 2015 position statement on the measurement of physical activity and provide guidance to clinicians and researchers for measuring physical activity in cystic fibrosis (CF) clinical practice and research.

**Methods:**

This study was registered with the International Prospective Register of Systematic Review (PROSPERO) database (CRD42022292165). Three databases (Medline, Embase and Cumulative Index to Nursing and Allied Health Literature) were searched for studies investigating the measurement of physical activity and sedentary time in people with CF irrespective of age or duration. The Quality Assessment for Diverse Studies was used to assess methodological concern. A mixed-methods framework synthesis was used to extract, map, chart, categorise and aggregate study findings.

**Results:**

In total, 7439 potentially relevant publications were identified. Following screening of titles and abstracts, 422 full texts were retrieved and assessed for eligibility, with 90 studies included. There was considerable variation in the methods of assessment, data processing and analytical interpretation of data.

**Conclusion:**

It is recommended that device-based physical activity metrics are presented as time spent in different intensity categories (*e.g.*, light, moderate and vigorous) and to include sedentary and sleep time. For data analysis, the data resolution should be at least 1 s (minimum 30 Hz) to enable clinical teams to obtain representative categorisation of patients’ physical activity patterns. Validated questionnaires (*e.g.*, the Habitual Activity Estimation Scale) offer additional opportunities to assess physical activity, whilst diaries can add context but should be viewed as secondary outcome measurements.

## Shareable abstract

**Device-based and validated physical activity questionnaires are recommended, inclusive of measuring sedentary and sleep time, to assess the importance of physical activity to the health and well-being of people with cystic fibrosis.** https://bit.ly/4jh9um9

## Introduction

In 2015, Bradley *et al*. [1], supported by the European Cystic Fibrosis Society (ECFS), published a position statement to inform the choice of physical activity assessment/measurement tools for cystic fibrosis (CF) research and clinical settings. Two physical activity monitors (SenseWear and ActiGraph) were endorsed for measuring energy expenditure (EE), step count, activity intensity and sedentary time [1]. The DigiWalker pedometer was noted as a low-cost alternative for assessing some physical activity components. Although several questionnaires were mentioned in the statement, only the Habitual Activity Estimation Scale (HAES) [2] was supported. Bradley *et al*. [1] concluded that “Future research should focus on providing additional evidence of clinimetric properties of these and new physical activity assessment tools, as well as further exploring the added value of physical activity assessment in CF”.

Since the 2015 position statement [1], research into physical activity measurement and interpretation of physical activity has increased substantially. Device-based assessment has advanced to include watch-worn, phone-based and clothing-embedded sensors, offering much higher data resolution than earlier tools [3]. High-resolution capture (≥100 Hz) enables analysis of activity in seconds rather than minutes [4], enhancing insight into the effects of varying intensities on health. Recently, physical activity has been conceptualised over a 24-h period, incorporating sedentary behaviour and sleep [5–7]. This has prompted the use of advanced statistical methods such as isotemporal substitution and compositional analysis [5–7].

In the general population, the type and volume of physical activity have a strong-to-moderate inverse association with morbidity and mortality [8]. In clinical groups, the importance of physical activity, even in those with complex conditions, is being increasingly recognised for physical, mental and social health benefits [9]. However, in CF, physical activity assessment is often not a routine clinical measure; with the transformational care and management of this disease and the latest cystic fibrosis transmembrane conductance regulator modulator drug therapies, the increased functional capability of this population warrants further investigation.

### Rationale

The recent advancements, not only in the measurement tools for physical activity but also conceptualisation and analyses, necessitate an update regarding the use of physical activity within CF research and clinical settings. Moreover, the shift towards conceptualising physical activity as a 24-h construct encompassing physical activity categories (light, moderate and vigorous), sedentary and sleep, has enabled a more comprehensive analysis of the role of physical activity in the lives of clinical groups. Indeed, new devices, methods of analysis and interpretation can help to advance the integration of physical activity in the management and care of people with CF (pwCF).

### Objectives

Therefore, the aim of this review was to systematically synthesise the literature on the measurement of physical activity, sedentary time and sleep in pwCF and provide guidance to clinicians and researchers.

### Review questions

This systematic review sought to address the following research questions:

 1) Which instruments (*i.e.* devices/questionnaires/diaries) represent the informed choice for the measurement of physical activity and sleep in clinical practice?
 2) Which instruments represent the informed choice for the measurement of physical activity and sleep as an outcome measure in research?
 3) What output(s) should be reported from device-based physical activity and sleep?
 4) What is an important treatment effect for device-based physical activity and sleep?
 5) What important consideration(s) should be made when collecting, processing and analysing device-based physical activity and sleep data in clinical practice and research?
 6) Specifically for device-based measurement of physical activity and sleep, what are the measurement and processing properties/features that should be standard for feasible utility in a clinical setting?

## Methods

This review was registered on the International Prospective Register of Systematic Review (PROSPERO) database (CRD42022292165) and conducted according to the Preferred Reporting items for Systematic Review and Meta-Analysis (PRISMA) [10, 11].

### Eligibility criteria

Studies investigating the measurement of physical activity, sedentary time and sleep, irrespective of age, setting and monitoring/study duration, were included. Peer-reviewed studies published in English were included independent of their design (*e.g.*, case studies and randomised controlled trials). Non-English-language, nonhuman and unpublished studies, book chapters, theses, dissertations, abstracts and monographs were not included. More specifically, studies investigating the association between the instrument used to measure physical activity with relevant health outcomes, such as lung function, body mass index (BMI) and quality of life, were included. Outcomes of any instrument that measures sedentary time and physical activity, either from devices or calculated from questionnaires (but excluding in-house nonvalidated questionnaires), but not limited to, min·week^−1^ of moderate physical activity, vigorous physical activity or/and sedentary time were reported.

### Information sources

A systematic search for published studies was conducted in March 2022 and updated in March 2024 using the Medline, Embase and Cumulative Index to Nursing and Allied Health Literature electronic databases. The reference list of relevant studies was examined for additional studies and experts in the field were contacted for any additional studies.

### Search strategy

As advised by the PRISMA checklist [10], a population–intervention–comparison–outcome framework was followed to structure the search strategy and a pilot search was performed to ensure the search strategy would be suitable. Limits and filters were applied to the search strategy for all databases to only include studies with humans that were published in English. As this review is not only updating the former ECFS position statement but expanding it, the year of publication was not restricted. The Medical Subject Headings (MeSH) were adopted to select the search terms, which were inserted as keywords into all three databases. The search terms were:

 1) Questionnaire* OR accelerometer* OR “motion sensor*” AND cystic fibrosis
 2) HAES OR IPAQ OR ActiGraph OR GENEActiv OR sensewear OR ActivPAL OR caltrac AND cystic fibrosis
 3) Physical activity OR habitual activity AND cystic fibrosis
 4) Sedentary time OR inactivity AND cystic fibrosis

### Selection process

The searches were imported into EndNote X9 (Clarivate Analytics, US) and subsequently into Rayyan [12] for screening. Two authors (M.S. Bianchim and C.A. Williams) double-blind screened all titles and abstracts, using Rayyan, according to the inclusion criteria. All discrepancies were resolved by consulting a third author to reach a consensus (J. Shelley, M.A. McNarry or K.A. Mackintosh). Subsequently, all full texts were saved as .pdf files and screened according to the pre-established criteria by the same authors (M.S. Bianchim and C.A. Williams), with a third author consulted if necessary (J. Shelley, M.A. McNarry or K.A. Mackintosh).

### Data collection process

A data extraction Excel spreadsheet was developed by two authors (M.S. Bianchim and C.A. Williams) based on preliminary data from the pilot and based on the main outcomes of interest. A total of seven reviewers (J. Shelley, M.A. McNarry, K.A. Mackintosh, T.J. Dwyer, B. Button, E. Hatziagorou and J. Bradley) collected data from each study independently using an online version of the spreadsheet. M.S. Bianchim resolved any disagreements and was responsible for managing the online data extraction spreadsheet making sure all data was extracted correctly. Supplementary information for each study was consulted when available or necessary and authors of the primary studies were consulted to obtain or confirm data.

### Data items

The main outcomes included those from instruments measuring physical activity, sedentary time and sleep (*e.g.*, minutes of moderate-to-vigorous physical activity, number of steps and EE (*e.g.*, metabolic equivalent tasks (METs)). Secondarily, additional outcomes included the different types of physical activity measures, along with their associated strengths and weaknesses (*e.g.*, participant compliance and differences between objective measures such as accelerometers and subjective measures such as questionnaires), any variables associated with collection, processing and analysis of physical activity data, and any data regarding the importance of physical activity and sedentary time measurement. Specifically, the following information was extracted from each included study: author, year of the study, sample information and characteristics (*e.g.*, age, size of the sample, BMI, stature, body mass and sex), any other health-related variable (*e.g.*, spirometry and heart rate), types of physical activity, duration and frequency of the protocol or intervention, and any variable related to collecting and processing of device-based physical activity (*e.g.*, epoch, sampling frequency, wear-time criteria and cut-points).

### Quality assessment

Each study was assessed independently by two reviewers (M.S. Bianchim, C.A. Williams, J. Shelley, M.A. McNarry, K.A. Mackintosh, T.J. Dwyer, B. Button, E. Hatziagorou or J. Bradley) using the Quality Assessment for Diverse Studies (QuADS) tool [13] and all disagreements were arbitrated by a third reviewer (M.S. Bianchim or C.A. Williams). The QuADS tool is composed of 13 domains and was specifically designed for the appraisal of mixed- or multi-methods studies, with strong inter-rater reliability (k=0.66) and substantial content validity [13]. The instrument was modified to assess the level of methodological concerns rather than the total numeric score [14]. Specifically, studies were rated as “no/minor”, “moderate”, “serious” or “very serious” methodological concerns. Findings from studies identified as serious and very serious concerns were interpreted with caution. Assessments were recorded using Microsoft Excel.

### Synthesis methods

A mixed-methods framework synthesis was used to extract, map, chart, categorise and aggregate study findings [15]. An initial framework was developed using the research questions as key domains from which to extract data. Initially, studies had key text of interest highlighted and then extracted into the initial framework (see supplementary file 1). Following the population of the framework with data, mapping and charting were undertaken using the mixed-methods data in its original format. The outcomes of this process were descriptive-level findings and explanations, which were shared and discussed with a wider group of researchers and key stakeholders.

## Results

**FIGURE 1:**
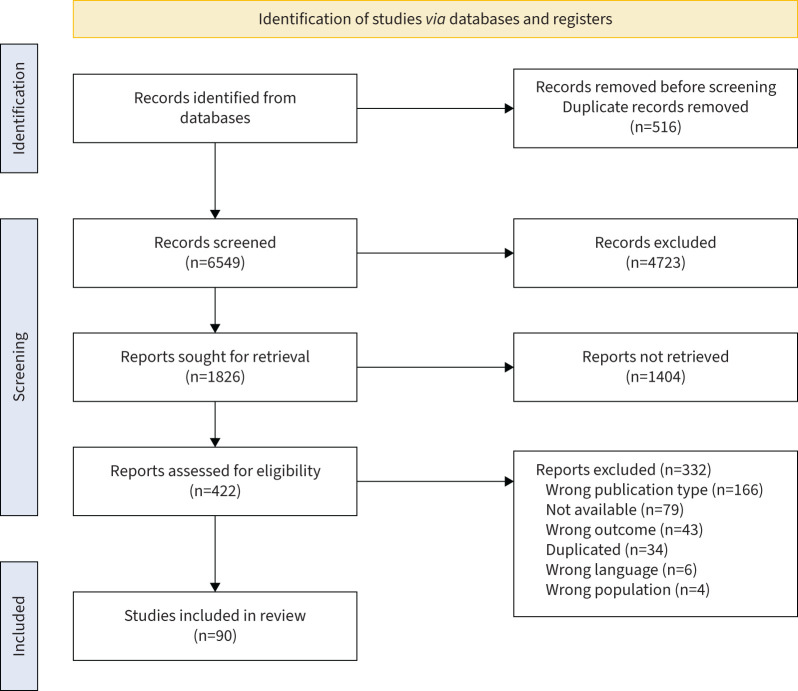
Flowchart of the study.

In total, 7065 potentially relevant publications were identified from the three databases, with 6549 remaining after removing duplicates. Following screening of titles and abstracts, 422 full texts were retrieved and assessed for eligibility leading to the exclusion of 332 studies, primarily due to wrong publication type (n=166) (figure 1). This resulted in the inclusion of 90 studies (table 1) with a total of 4021 participants with CF aged between 6.7 and 65.0 years (mean±sd 21.1±17.4 years) and 48.9% were male (n=1968). 51 of the included studies were published after the 2015 position statement. All excluded studies are listed in supplementary file 2. 36 studies were in adults [16–51], 34 in young people [52–84] (27 included children, five children and adolescents, and two adolescents only) and 20 included both young people and adults [85–103]. Only 34 studies included a control group (n=5934) [16, 23, 26, 27, 30, 37, 43–45, 48, 52–54, 57, 59, 61, 62, 66, 68, 70, 72, 74, 75, 79–81, 91, 92, 94, 95, 98–100, 104]. The included studies measured physical activity using accelerometers (n=57) [16–38, 41, 50, 52–56, 58–69, 72, 86–93, 103–107], questionnaires (n=26) [40, 42, 43, 45, 47, 48, 51, 70–77, 79–84, 95, 96, 98–100], both accelerometers and questionnaires (n=11) [21, 26, 53, 54, 56, 60, 72, 87, 88, 92, 103], diaries (n=8) [58, 59, 78, 83, 88, 89, 96, 108], commercial smartwatches (n=5) (Garmin Vivosmart4® and Fitbit® Charge 2) [19, 41, 56, 94, 97], pedometers (n=3) [49, 101, 102] and interviews (n=1) [109].

**TABLE 1:** Characteristics of the included studies

Study, year	Characteristics
**Anifanti *et al.* [94] 2022**	
Methods	Design: RCT Inclusion criteria: patients with a stable CF clinical status, without worsening respiratory symptoms or overt pulmonary hypertension, or a history of contemporary heart issues Exclusion criteria: patients already participating in structured PA
Participants	42 participants with CF 23 males, aged 16.8±3.6 years Group A (n=21) mean±sd age 17.0±3.3 years, BMI 20.0±3.3 kg·m^−2^ Group B (n=21) mean±sd age 16.1±3.3 years, BMI 20.1±2.6 kg·m^−2^
Intervention	Randomly selected into intervention (group A) or control (group B) groups Group A participated in a 1-year WAT-based ET programme three times per week All patients underwent a 6MWT and an echocardiographic assessment focused mainly on RV anatomy and function at the baseline and the end of the study RV systolic function was evaluated and RV diastolic function was assessed PASP was also estimated
Outcomes	Functional capacity assessment (6MWT), echocardiographic studies and step count
Notes	A G1 (Garmin) WAT was used for the assessment of physical activity
**Aznar *et al.* [52] 2014**	
Methods	Design: cross-sectional Inclusion criteria: outpatient diagnosed using a genetic test for CF and treated at hospital, and boys/girls aged 6–17 years living in the Madrid region Exclusion criteria: having severe lung deterioration, an unstable clinical condition, *Burkholderia cepacia* infection or any condition impairing exercise testing
Participants	47 participants with CF, 39 control subjects 6–17-year-old CF patients PA group (n=47) mean±sd age 12±3 years Control group (n=39) mean±sd age 12±2 years
Intervention	PA measured across 7 days on participant's right hip during the day *V*′_O_2___peak_ measured on a treadmill
Outcomes	This study examined *V*′_O_2___peak_, (mL·kg^−1^·min^−1)^, SED (min·day^−1^), total PA (min·day^−1^), light PA (min·day^−1^), MVPA (min·day^−1^), vigorous PA (min·day^−1^)
Notes	The assessment of PA used an Actigraph GT3X
**Béghin *et al.* [108] 2003**	
Methods	Design: RCT Inclusion criteria: CF patients aged 5–18 years, chronically colonised with *Pseudomonas aeruginosa* Exclusion criteria: cardiac insufficiency, cardiac rhythm abnormalities, treatment with beta-blockers, oxygen therapy and lung transplantation; in addition, if patients required hospitalisation, treatment with corticosteroids or presented with any acute condition known to interfere with EE
Participants	16 participants with CF PA group (n=16) mean±sd age 12.1±2.1 years Control group not included
Intervention	Each subject was studied twice, 3–7 days before administration of IVAT and 5–10 days after completion of 14 days IVAT HR and PA measured on a day without sporting activity Food record, body composition, REE and the calibration equation for HR and EE were again assessed, mean±sd 28±4 days after the first evaluation The following day, HR and PA were simultaneously recorded for 24 h on the same school day of the week as the first evaluation
Outcomes	This study examined *V*′_O_2__ at rest (L·min^−1^), *V*′_CO_2__ at rest (L·min^−1^), RQ at rest, TEE (kJ·day^−1^), TEE/REE and PA (MET·day^−1^)
Notes	This study was conducted in the Clinical Research Center of Lille, University Hospital (CIC-9301-INSERM-CHU).
**Béghin *et al.* [60] 2005**	
Methods	Design: observational study Inclusion criteria: children and adolescents with CF who were chronically colonised with *P. aeruginosa* and needed a semi-elective IVAT when the Smith score was more than 5/10
Participants	16 participants with CF, nine boys and seven girls, median weight of 33.7 kg (range 20.5–46.8 kg), FFM of 26.5% (18.0–41.7%) and height of 141 cm (range 118–157 cm) PA group (n=16) median age 12.1 (range 7.1–14.6) years
Intervention	Each subject was studied twice before and after IVAT both in laboratory conditions in hospital and in free-living conditions during a school day Each evaluation was carried out 3–7 days before the administration of IVAT and 5–10 days after the completion of a 14-day IVAT
Outcomes	This study examined PA (counts·min^−1^), DEE (kJ·min^−1^·kg^−1^ FFM), PAEE (kJ·min^−1^·kg^−1^ FFM), PAEE/PA (kJ·min^−1^·kg^−1^ FM/counts), forced vital capacity, FEV_1_, PEF and FEF_25–75%_
Notes	This study was carried out in the Clinical Research Center of Lille, University Heart Hospital (CIC-9301-INSERM-CHU)
**Bianchim *et al.* [93] 2022**	
Methods	Design: compositional analysis Exclusion criteria: participants with multi-resistant bacteria (*e.g.*, *B. cepacia* complex and nontuberculous mycobacteria), comorbidities that might compromise being physically active (*e.g.*, cardiovascular and musculoskeletal) or who were awaiting a transplant
Participants	129 participants with CF 86 children (45 boys) and 43 adults (22 males) Children group (n=86) mean±sd age 13.6±2.8 years, BMI 18.7±3.4 kg·m^−2^ Adult group (n=43) mean±sd age 24.6±3.5 years, BMI 21.2±4.4 kg·m^−2^
Intervention	Wrist-worn accelerometery was used to assess PA, SED and sleep Compositional linear regression models were conducted following normalisation *via* isometric log-ratio transformations
Outcomes	This study examined SED, LPA, MVPA and sleep
Notes	This study noted the importance of prioritising sleep as reductions in sleep were associated with detrimental effects in lung function
**Bianchim *et al.* [69] 2022**	
Methods	Design: RCT Inclusion criteria: aged 7–18 years diagnosed as having CF through a newborn screening test and/or those presenting CF-typical symptoms and either two pathological sweat tests or the identification of two CF-relevant mutations Exclusion criteria: the presence of multi-resistant bacteria or being on the transplant list
Participants	71 participants with CF 35 boys; 46 participants had mild CF, 24 had moderate CF and one had severe CF Mean±sd age 13.5±2.9 years, BMI 19.0±3.9 kg·m^−2^
Intervention	PA was assessed for 7 consecutive days using a nondominant wrist-worn ActiGraph GT9X CF-specific and generic ENMO cut-points were used to determine SED, sleep, LPA, MPA and VPA The effect of using a CF-specific or generic cut-point on the relationship between PA intensities and lung function was determined
Outcomes	This study examined SED, LPA, MPA, VPA, MVPA and sleep
Notes	Aim to compare the use of generic and CF-specific cut-points to assess movement behaviours in children and adolescents with CF
**Boni *et al.* [39] 2022**	
Methods	Design: retrospective study Exclusion criteria: patients who undergone lung transplantation and patients who have started, in the same period, any CFTR modulator therapies (*i.e.*, IVA, LUM, elexcaftor, tezacaftor or their combinations)
Participants	111 participants with CF 57 males Median age 35 (IQR 24–44) years
Intervention	Retrospectively reviewed clinical data, according to two periods: pre-lockdown (from October 2019–March 2020) and post-lockdown (from May 2020–October 2020) Data on nutritional (BMI and body weight) and lung function status; was collected Patients were divided into three groups, according to FEV_1_ value, as follows: group 1 (FEV_1_ <40%), group 2 (FEV_1_ 40–70%), group 3 (FEV_1_ >70%) All patients received a telephone interview asking for the number of hours per week devoted to PA, number of pulmonary acute exacerbations and subjective evaluation of adherence to medical therapy, respiratory physiotherapy and diet during the two periods
Outcomes	This study examined weight, BMI, respiratory function and amount of PA
Notes	This study examined the effect of lockdown during the COVID-19 pandemic and reported improvements in weight and some stabilisation and improvement of lung function in some patients with CF
**Britto *et al.* [70] 2000**	
Methods	Design: retrospective study Inclusion criteria: adolescents with CF identified through active patient registries of both North Carolina Cystic Fibrosis Care Centers Exclusion criteria: if no demographic match existed
Participants	115 participants with CF, 59 females, 56 males, 93.9% White, 3.5% Black and 2.6% other PA group (n=115) median age 15 years, ≤14 years (n=48), 15–16 years (n=31), ≥17 years (n=36)
Intervention	PA was assessed using questions from the Centers for Disease Control's Youth Risk Behaviour Survey
Outcomes	This study examined VPA, participation in school or community sports teams, participation in physical education classes, and global health status (CF group only)
Notes	This work was prepared in conjunction with the Division of Innovation and development Services of the North Carolina Department of Public Instruction
**Buntain *et al.* [95] 2004**	
Methods	Design: controlled cross-sectional study Inclusion criteria: aged 5 years or older attending the Royal Children's Hospital or the Adult clinic at The Prince Charles Hospital, Brisbane Exclusion criteria: Patients who have had a lung transplant or had primary bone disease, a chronic illness known to affect bone density or a period of immobility of more than 2 weeks in the preceding 12 months
Participants	153 participants with CF PA group (n=153) mean±sd age 13.6±0.27 years Control group (n=149) mean±sd age 13.3±0.20 years
Intervention	Bone densitometry measurements of TB, LS, femoral neck, cortical radius and ultra distal radius measured using DEXA Respiratory function tests were performed, food intake diaries were taken and PA questionnaires for older children used Random nonfasting blood samples were collected
Outcomes	This study examined BMD, vitamin D status, PA, dietary intake, corticosteroid exposure, fracture incidence and associations with BMD in individuals with CF
Notes	The study was supported by Cystic Fibrosis Research Australia Pty Ltd and the Royal Children's Hospital Foundation
**Buntain *et al.* [74] 2006**	
Methods	Design: controlled longitudinal study Inclusion criteria: confirmed diagnosis of CF *via* an elevated sweat chloride test Exclusion criteria: had a primary bone disorder or awaiting lung transplantation
Participants	40 participants with CF Children group age 5–10 years, adolescent group age 11–18 years PA children group (n=40) mean±sd age 8.5±1.8 years, 19 males PA adolescents group (n=45) mean±sd age 14.1±2.2 years, 27 males Control children group (n=32) mean±sd age 8.5±1.5 years, 14 males Control adolescents group (n=68) mean±sd age 13.9±1.9 years, 32 males
Intervention	Areal BMD of the TB, LS and total femoral neck were repeatedly measured in 85 subjects aged 5–18 years with CF and 100 age- and sex-matched controls over 2 years At each visit, anthropometric variables, nutritional parameters, pubertal status, disease severity, PA, dietary calcium, caloric intake, and serum 25OHD were assessed and related to areal BMD
Outcomes	This study examined BMD, corticosteroid exposure and fracture incidence
Notes	This study examining bone density used DXA
**Burghard *et al.* [71] 2022**	
Methods	Design: cross-sectional study Inclusion criteria: no ventilatory limitation during exercise and free from acute pulmonary exacerbation at the time of testing
Participants	60 participants with CF 51.7% male Impaired glucose tolerance including CFRD mean±sd age 12.5±2.7 years CFRLD mean±sd age 8.3±3.1 years
Intervention	CPET was used to determine *V*′_O_2___peak_ normalised to body weight as a measure of CRF Patients were defined as having “low CRF” when CRF was less than 82% pred Self-reported PA data and pulmonary function data were also collected
Outcomes	This study examined mean CRF, CFRLD, IGT including CFRD, *P. aeruginosa* colonisation*,* pulmonary function, *V*′_O_2___peak_ (% pred), sweat chloride concentration and CFTR-modulating therapies
Notes	Data presented earlier at the European Cystic Fibrosis Society Conference 2021 (www.ecfs.eu/digital2021)
**Burnett *et al.* [40] 2020**	
Methods	Design: cross-sectional, descriptive study Inclusion criteria: patients with CF, 18 years old and able to communicate in English Exclusion criteria: not reported
Participants	46 participants with CF 52% male, 42 White ethnicity, three black or African American ethnicity and one Hispanic or Latino Mean±sd age 31±10.6 years
Intervention	7-day PA recall questionnaire, Twin City Walking Survey
Outcomes	This study examined PA level, exercise preference, exercise readiness and exercise barriers
Notes	A 7-day PA recall questionnaire during a structured interviewed assessed activity levels
**Burtin *et al.* [37] 2013**	
Methods	Design: prospective case–control study Inclusion criteria: patients with four or more symptoms of exacerbations Exclusion criteria: the presence of orthopaedic conditions interfering with the assessment of skeletal muscle force, the occurrence of a life-threatening exacerbation, an operation in the inguinal region in the previous 2 months (contra-indication to perform magnetic stimulation) and inclusion in a structured ET programme
Participants	19 participants with CF 13 males and six females with CF and exacerbations, six males, four females; “stable” patients PA group (n=19) mean±sd age 25±6 years Control group (n=10) mean±sd age 29±8 years
Intervention	Muscle strength assessment, spirometry and venous blood sampling were performed at the beginning (day 1) and at the end of IVAT (day 14) and 1 month after IVAT (day 40±3 days) 6MWD was assessed at day 14 and day 40 PA levels were measured using activity monitoring during (from day 1 to day 14) and 1 month after the exacerbation (2 weeks starting from day 40)
Outcomes	This study examined assessment of quadriceps strength, PA, spirometry, 6MWD and blood analysis
Notes	This study used a SenseWear Pro Armband to quantify PA
**Burton *et al.* [38] 2020**	
Methods	Design: single-centre prospective cohort observational study Inclusion criteria: adults with a formal diagnosis of CF (aged 18 years and above) who were admitted for inpatient hospital treatment for a pulmonary exacerbation Exclusion criteria: <18 years at the time of enrolment, discharged with home-based IVAT or were pregnant
Participants	31 participants with CF PA group (n=31) median age 28.8 (range 18–62) years
Intervention	CF patients enrolled at the end of an inpatient admission at The Prince Charles Hospital, Brisbane Discharge determined by self-reported improvement in patient's symptoms and objective evidence of a reduction in CRP and an improvement in lung parameters PA post-discharge measured by SenseWear armband for 5–7 days To monitor time to next pulmonary exacerbation (days), all participants were followed-up for 12 months
Outcomes	This study examined sputum supernatant and plasma concentration of IL-6, IL-8 and TNF-α Daily average metabolic equivalents, duration of PA at a moderate-high level and daily average steps collected using the SenseWear armband Time to pulmonary exacerbation (days)
Notes	This study was registered with Physiotherapy Theory and Practice
**Campos *et al.* [72] 2020**	
Methods	Design: cross-sectional study CF group inclusion criteria: patients with confirmed clinical diagnosis of CF (sweat test or genetic test) and presenting with stable clinical state at the day of assessment Control group inclusion criteria: healthy individuals, based on a respiratory health questionnaire review and lung function analysis Exclusion criteria: any individuals who failed to perform any of the tests in the study
Participants	30 participants with CF, 25 control participants 63.3% male in CF group, 52% male in control group PA group (n=30) mean±sd age 16.9±5.1 years Control group (n=25) mean±sd age 16.2±5.0 years
Intervention	A questionnaire assessing PA level and video games played on two consoles with 10-min intervals to assess physiological variables Accelerometer used to measure PA level during tests
Outcomes	This study examined exercise capacity values for resting, anaerobic threshold and peak exercise periods, HR, ventilation, *V*′_O_2__ and metabolic demand data
Notes	This study was registered as number NCT03229213 (www.clinicaltrials.gov).
**Causer *et al.* [56] 2022**	
Methods	Design: case series Inclusion criteria: required to have the time to complete the study, be within reasonable distance of the centre for teachers to travel to lessons and have internet access Exclusion criteria: if participated in any other interventional study or if they had participated in the pilot study
Participants	Three participants with CF Age range 13.1–15.7 years, two males
Intervention	Participants completed an exhaustive maximal cardiopulmonary exercise test on a cycle ergometer to determine *V*′_O_2___peak_ and measure changes in gas exchange and ventilation during exercise at 6 weeks Analysed wrist-worn device-based PA data in two of the three cases; validated acceleration thresholds were used to quantify time spent in each PA intensity category
Outcomes	This study examined FVC, FEV_1_, oxygen saturation, BMI, gas exchange, total PA and *V*′_O_2___peak_
Notes	In two of the three case studies a GENEActiv accelerometer was used to measure PA
**Conway *et al.* [96] 2000**	
Methods	Design: cross-sectional study Inclusion criteria: CF confirmed by identification of two CF gene mutations or by two diagnostic sweat tests Exclusion criteria: none
Participants	114 participants with CF 37 adolescents (16 males, mean±sd age 18.7±0.8 years; 21 females, mean age 18.4±1.0 years) 77 adults (37 males, mean±sd age 26.6±5.6 years; 40 females, mean age 28.2±5.7 years) 66 (58%) were homozygous, 31% heterozygous for the delta F508 mutation 35% had diabetes mellitus and 54% had CFRLD 89% were chronically infected with *P. aeruginosa* and 10% with *B. cepacia*
Intervention	BMD measurements made at LS (L2–L4), right femoral neck and TB Blood was sampled for measurement of liver function tests, calcium, phosphate, 25-OH vitamin D, follicle-stimulating hormone, luteinising hormone, oestradiol (females), testosterone (males) and thyroid function Participants completed a 4-day unweighed diet and enzyme diary Exercise recalled using 7-day activity recall questionnaire
Outcomes	This study examined BMD measurements, blood samples and nutritional data
Notes	Adult patients with CF showed a high prevalence of vitamin D insufficiency
**Cox *et al.* [34] 2014**	
Methods	Design: cross-sectional study Inclusion criteria: >18 years, with stable CF Exclusion criteria: comorbidities that limited PA participation, current use of *i.v*. antibiotics or long-term oxygen therapy, pregnancy, or lung transplantation
Participants	26 participants with CF 11 males, mean±sd BMI 22±2 kg·m^−2^, height 168±8 cm Mean±sd age 28±7 years
Intervention	Participants completed seven PA tasks with simultaneous assessment of EE, a mix of static tasks and active tasks
Outcomes	This study examined EE and exhaled respiratory gases
Notes	EE from the SenseWear armband resulted in a moderate degree of agreement with EE measurements from indirect calorimetry
**Cox *et al.* [33] 2015**	
Methods	Design: cross-sectional study Inclusion criteria: confirmed diagnosis of CF Exclusion criteria: comorbidities that limited mobilisation or participation in PA, prior lung transplantation, pregnancy, or lack of home internet access
Participants	10 participants with CF Four males Mean±sd age 30±8 years All subjects were pancreatic insufficient
Intervention	Each subject logged into ActivOnline a mean±sd of 13±11 times over the 8-week intervention period Programme provided users with real-time graphical representation of their PA entries (including duration and step count) A pedometer was provided to monitor step count
Outcomes	Feasibility of software to encourage activity participation assessed by frequency of site access, number of activity sessions recorded and number of telephone consultations undertaken Acceptability of software assessed through interviews and subject preference ratings using Likert scales Secondary outcome variables: PA participation, HRQoL and exercise capacity
Notes	This study utilised a specifically designed internet-based PA programme
**Cox *et al.* [35] 2016**	
Methods	Design: observational study Inclusion criteria: adults attending two specialist CF centres in Melbourne, Australia, with a confirmed diagnosis of CF, aged ≥18 years Exclusion criteria: *i.v.* antibiotics for a respiratory exacerbation in the 4 weeks preceding baseline assessment; comorbidities limiting mobilisation or PA participation, colonisation of respiratory secretions with *B. cepacia*, pregnancy, or lung transplant recipient
Participants	65 participants with CF 34 males, mean±sd age 28±8 years, BMI 22±3 kg·m^−2^
Intervention	PA monitoring at baseline and after 12 months Measured over 5–7 days using a portable multi-sensor armband
Outcomes	This study examined PA, pulmonary function, exercise capacity and CF-related QoL assessed during a stable phase
Notes	This study was registered as ACTRN12610000949088 at the Australian New Zealand Clinical Trials Registry
**Cox *et al.* [36] 2019**	
Methods	Design: observational study Inclusion criteria: adults attending two specialist CF centres in Melbourne, Australia, with a confirmed diagnosis of CF, aged ≥18 years Exclusion criteria: *i.v.* antibiotics for a respiratory exacerbation in the 4 weeks preceding baseline assessment, comorbidities limiting mobilisation or PA participation, colonisation of respiratory secretions with *B. cepacia*, pregnancy, or lung transplant recipient
Participants	65 participants with CF 34 males, mean±sd age 28±8 years, BMI 22±3 kg·m^−2^
Intervention	PA monitoring at baseline and after 12 months Measured over 5–7 days using a portable multi-sensor armband
Outcomes	This study examined PA, pulmonary function, exercise capacity and CF-related QoL assessed during a stable phase
Notes	This study is a secondary analysis of Cox *et al.* **[36]**
**Cox *et al.* [92] 2022**	
Methods	Design: RCT Inclusion criteria: confirmed diagnosis of CF, aged 12–35 years (inclusive) and access to the internet *via* a computer or mobile device Exclusion criteria: severe comorbidity limiting mobilisation or PA participation (*e.g.* orthopaedic, cardiac or neurological condition), recipient of a lung transplant, pregnancy, or they (or their parents) are unable to provide informed consent
Participants	107 participants with CF ActivOnline intervention group (n=52): mean±sd age 21±7 years, 24 male, BMI 21±3 kg·m^−2^ Usual care control group (n=55): mean±sd age 20±6 years, 23 male, BMI 21±3 kg·m^−2^
Intervention	All participants received usual care and were provided with information, *via* a web link, on age-appropriate recommendations for being physically active In addition, participants randomised to the intervention (ActivOnline) group were provided with individualised access to a secure web platform The web platform was used to record and monitor PA, and set goals, for the 12-week intervention period Data entered were updated in real time and feedback presented in a graphical display
Outcomes	This study examined change in device-based average daily MVPA from baseline to the end of the 12-week intervention period, measures of PA (self-reported), self-determination for exercise, HRQoL, psychological well-being, exercise capacity (MST) and lung function
Notes	This study was registered as ACTRN12617001009303 at the Australian New Zealand Clinical Trials Registry
**Curran *et al.* [19] 2021**	
Methods	Design: observational study Inclusion criteria: required to have a confirmed diagnosis of CF and be able to walk unaided for at least 40 min Exclusion criteria: any neurological disorder, cognitive disorder or musculoskeletal injury that would impair walking
Participants	21 participants with CF Six males, mean±sd BMI 21.7±3.3 kg·m^−2^ PA group (n=21) mean±sd age 25.3±5.98 years
Intervention	Participants walked on a treadmill for 5 min at five pre-determined speeds and at three self-determined speeds along a corridor, accelerometers were worn
Outcomes	This study examined observer step count and device step count (×2)
Notes	This work was supported by Truck Run 4 Katie, the Health Research Institute and the University of Limerick
**Curran *et al.* [41] 2022**	
Methods	Design: observational study Inclusion criteria: aged 18 years or older, confirmed diagnosis of CF and clinically stable CF (*i.e.*, not experienced a pulmonary exacerbation in the last month) Exclusion criteria: FEV_1_ <25% pred, patients on the waiting list for lung transplantation or prior lung transplantation, patients dependent on supplemental oxygen for exercise, pregnancy, any cardiac, neurological or musculoskeletal impairment that impacted ability to be physically active, in another clinical trial up to 4 weeks prior to their PA assessment, and patients with an exacerbation in the 4 weeks prior to the study
Participants	33 participants with CF 61% female, mean age 26.2±7.1 years
Intervention	Participants were instructed to wear an accelerometer continually for 7 days, at the end of which, the participants returned to an adult CF unit, for exercise testing, spirometry and completion of questionnaires CPET was conducted after a period of rest
Outcomes	This study examined PA, aerobic capacity, sleep and well-being
Notes	This observational study design followed the STROBE standardised reporting guidelines to conduct and report the study
**Curran *et al.* [104] 2022**	
Methods	Design: pilot randomised trial Inclusion criteria: age ≥18 years, confirmed diagnosis of CF and clinically stable who were not experiencing a pulmonary exacerbation Exclusion criteria: FEV_1_ <25%, on the waiting list for lung transplantation or had undergone lung transplantation, respiratory exacerbation in the 4 weeks prior to the study, dependency on supplemental oxygen for exercise, current pregnancy, any cardiac, neurological or musculoskeletal impairment that impacted ability to engage in a PA intervention, or participation in another clinical trial up to 4 weeks prior to the first baseline visit
Participants	33 participants with CF Intervention group (n=17): mean±sd age 26.7±7.8 years, five males, BMI 21.5±2.2 kg·m^−2^ Active comparator group (n=16): mean±sd age 24.5±5.4 years, eight males, BMI 23.2±4.1 kg·m^−2^
Intervention	The 12-week intervention consisted of technology (Fitbit Charge 2) which was remotely monitored and participants set step count goals Participants were sent a one-way text message once a week over 12 weeks to positively reinforce and encourage PA participation The active comparator group received the wearable technology alone Follow-up was assessed at 24 weeks
Outcomes	This study examined PA, aerobic capacity, lung function, sleep, QoL and well-being
Notes	The study protocol is registered on ClinicalTrials.gov (NCT03672058)
**Currie *et al.* [42] 2017**	
Methods	Design: cross-sectional study Inclusion criteria: aged 18 years or older and had documented diagnoses of CF and CFRD in their medical record Exclusion criteria: liver, lung or kidney transplant recipient, unable to speak or understand English, use of *i.v.* antibiotics or corticosteroid therapy (*i.v.* or oral) within 3 months of recruitment, documented liver disease, pregnancy, or an inpatient at the time of the study
Participants	18 participants with CF Mean±sd age 41±9 years, 10 males, BMI 23.2±2.5 kg·m^−2^
Intervention	Adults with CFRD were recruited from a hospital-based CF clinic PA was measured using 7-day PA recall (telephone interview), adherence to CFRD management with the Self-Care Inventory–Revised (questionnaire) and blood glucose control from glycated haemoglobin levels documented in participants’ medical charts within 3 months
Outcomes	This study examined amount of time spent in different types of PA
Notes	First study to describe PA levels in individuals with CFRD
**Dassios *et al.* [73] 2022**	
Methods	Design: secondary analysis of a study cohort Exclusion criteria: any children or young people with a pulmonary exacerbation in the past 2 weeks or acute illness and hospitalisation
Participants	28 participants with CF Median age 15 (IQR 13–17) years, 11 males
Intervention	FFM index measured using bioelectrical impedance, lung function using spirometry, number of shuttles as a measure of exercise tolerance and the reported PA in children
Outcomes	This study examined FFM, BMI, pulmonary function and respiratory muscle testing, HAES and the MST
Notes	The HAES assessed inactive and active periods of the day and included documentation of waking, sleeping and mealtimes
**De Freitas Coelho *et al.* [63] 2022**	
Methods	Design: cross-sectional study Inclusion criteria: children and adolescents with a diagnosis of CF, in stable clinical conditions (no signs of pulmonary exacerbation in the previous 3 months), without heart disease and who were able to fully comprehend all evaluations performed Exclusion criteria: individuals who failed to complete all tests
Participants	30 participants with CF 66.6% female, mean±sd age 11.2±3.7 years Majority had a heterozygous F508del genotype (43.3%) and were colonised by *Staphylococcus aureus* (63.3%) Pancreatic insufficiency was present in 86.4% of participants
Intervention	During a consultation, a spirometry test was undertaken; then, participants were referred to perform an HRV assessment using a cardiofrequency meter The evaluation was performed during 25 min at rest and during the MST Triaxial accelerometer used for a period of 5 days
Outcomes	This study examined lung function, levels of daily activity, resting and peak exercise values
Notes	This study noted a sympathetic HRV predominance and normal physiological exercise response in children and adolescents with mild-to-moderate CF
**Decorte *et al.* [43] 2017**	
Methods	Design: RCT Exclusion criteria: clinically unstable, contraindications for maximal exercise testing or severe limb joint condition, FEV_1_ <40%; receiving long-term oxygen therapy or corticotherapy
Participants	15 participants with CF 12 males, three females with CF, same as controls Mean±sd BMI 21.4±7.5 kg·m^−2^ for CF group, BMI 22.2±2.6 kg·m^−2^ for control group PA group (n=15) mean±sd age 28.1±6.2 years Control group (n=15) mean±sd age 26.5±4.6 years
Intervention	Participants performed a maximal CPET and two localised calf muscle tests (maximal incremental and constant-load) using magnetic resonance spectroscopy Habitual PA and QoL assessed by questionnaires
Outcomes	This study examined baseline characteristics, muscle energetic kinetics during and after calf exercises, maximal incremental plantar flexion test, and constant load plantar flexion test
Notes	The only study to measure phosphocreatine recovery, an indicator of maximal oxidative capacity and PA
**Dhillon *et al.* [18] 2018**	
Methods	Design: method comparison study Inclusion criteria: adult participants (between 35 and 65 years) with clinically diagnosed COPD or interstitial lung disease, and adult participants (19 years and older) with clinically diagnosed CF; had to be clinically stable and medically cleared for CPET Exclusion criteria: patients with previously transplanted organs, requiring mobility aids to ambulate or supplemental oxygen during exercise, who were colonised with *B. cepacia* complex, *Myobacterium abscessus* and/or *B. dolosa* and closely related organisms (*Pandoraea* *apista* and *Ralstonia* spp.) were excluded for infection prevention and control reasons Patients with poor adherence to medications, uncontrolled diabetes, profound emaciation (BMI <16 kg·m^−2^) or indices of pulmonary hypertension were excluded
Participants	Five participants with CF Age range 23–65 years, BMI range 18–31 kg·m^−2^
Intervention	Spirometry testing was performed, as was a standardised incremental cycle ergometer Flex HR method calibration testing and PA measurement
Outcomes	This study examined EE, *V*′_O_2___peak_, HR_peak_ and indirect calorimetry
Notes	This study used both the SenseWear armband and an Actical accelerometer to assess PA
**Dietz-Terjung *et al.* [105] 2021**	
Methods	Design: sub-analysis of a previous study Inclusion criteria: confirmed diagnosis of CF Exclusion criteria: patients with known cor pulmonale, musculoskeletal diseases that do not allow continuous ET and patients with an untreated or poorly adjusted CF-related diabetes mellitus Patients with uncompleted data sets were also excluded
Participants	109 participants with CF 59% male, mean±sd BMI 19.4±3.4 kg·m^−2^, mean±sd age 22.7±12.0 years
Intervention	Sleep monitor worn for 4 weeks on the nondominant hand
Outcomes	This study examined steps·day^–1^, amount of sedentary/light/moderate/vigorous/very vigorous exercise, sleep efficiency, time in bed, total sleep time and waking after sleep onset
Notes	Sub-analysis of the CFmobil project, a 12-month partially supervised exercise programme for CF patients ≥6 years of age
**Dobbin *et al.* [44] 2005**	
Methods	Design: case–control study Inclusion criteria: older than 16 years with CF
Participants	22 participants with CF CF group (n=22) mean±sd age 26±9 years, 36% male, BMI 20±2 kg·m^−2^ Control group (n=22) mean±sd age 30±8 years, 64% male, BMI 22±4 kg·m^−2^
Intervention	Participants completed a quality-of-life questionnaire, underwent polysomnography while breathing room air with evening and early-morning arterial blood gases, also on room air, and had neurobehavioral testing, before and after inpatient treatment of an exacerbation Neurobehavioral testing was performed either in the afternoon before or on the morning after polysomnography 12 cases had neurobehavioral testing performed after spending their first night in hospital Subjects were tested at the same time of day on each occasion to control for circadian variation in vigilance and alertness Total sleep time minimum average *S*_pO_2__ was calculated by averaging the lowest saturation measurement in each 30-s period of sleep Intelligence was assessed using the Shipley Institute of Living Scale Spirometry, resting daytime *S*_pO_2__ and weight were measured before each test bout
Outcomes	This study examined sleep architecture, gas exchange and the Neurobehavioral Assessment Battery
Notes	This study demonstrated that exacerbations of lung disease in young adults with CF adversely affected sleep and neurobehavioral performance, irrespective of disease severity
**Dwyer *et al.* [16] 2009**	
Methods	Design: Evaluation of Accuracy of SenseWear Activity Monitor Inclusion criteria: confirmed diagnosis of CF Exclusion criteria: received a lung transplant, colonised with *B. cepacia*, not clinically stable or unable to exercise for 20 min without stopping
Participants	17 participants with CF CF group (n=17) mean±sd age 26±6 years, six males Control group (n=17) mean±sd age 29±7 years, nine males
Intervention	Spirometry testing prior to exercising Subjects walked on a motorised treadmill for 20 min without slowing/stopping, whilst simultaneously wearing an activity monitor and breathing through an indirect calorimetry system For 10 min, there was 0% incline; after 10 min, a 5% and 10% incline for the CF and control subjects, respectively
Outcomes	This study examined exercise intensity, EE and step count
Notes	The SenseWear armband significantly underestimated EE at higher exercise intensities
**Elmesmari *et al.* [64] 2022**	
Methods	Design: case–control study Inclusion criteria for CF patients: attending the outpatient clinic at the Royal Hospital for Children (Glasgow, UK), previously diagnosed with CF, between 3 years and 10 years of age, with no limitations to walking or PA because of other mobility issues Exclusion criteria: had an orthopaedic condition limiting PA, using a wheelchair for mobility or with another condition that may impact their PA or younger than 3 years and older than 10 years of age
Participants	20 participants with CF Mean±sd age 6.7±2.0, eight males, BMI 17.3±3.9 kg·m^−2^
Intervention	Accelerometer was used for a period of 7 consecutive days.
Outcomes	This study examined time spent sitting, number of sitting bouts, time standing, time in PA, steps per 24 h, time spent sleeping, number of participants not meeting steps recommendation and not meeting sleep recommendation
Notes	The activPAL micro monitor was used to measure PA
**Enright *et al.* [45] 2007**	
Methods	Design: observational, case–control study Inclusion criteria: confirmed CF Exclusion criteria: liver cirrhosis, cor pulmonale or an exacerbation of their respiratory symptoms (increased respiratory symptoms, weight loss, fever or reduction in FEV_1_ of >10% than the usual value) or elevated CRP (>15.5 μg·mL^−1^)
Participants	40 participants with CF, 22 males CF patients with low FFM (n=22) mean age 22.3 (range 20.7–23.9) years, BMI 21.5 (range 20.7–22.3) kg·m^−2^ CF patients with normal FFM (n=18) mean age 22.6 (range 20.6–24.6) years, BMI 22.4 (range 21–23.8) kg·m^−2^ Healthy subjects (n=30) mean age 21.7 (range 20.3–23.1) years, BMI 23 (range 22.2–23.8) kg·m^−2^
Intervention	Body composition, pulmonary function and PA status were determined at initial screening DXA scans taken and diaphragm thickness measured Dry wedge spirometry taken to measure lung function Circulating CRP determined and a recall physical activity questionnaire filled out Inspiratory muscle function measured
Outcomes	This study examined body composition, pulmonary function, PA status, FFM, diaphragm thickness, lung function, circulating CRP, PA and inspiratory muscle function
Notes	A loss of FFM coupled with worsening pulmonary disease resulted in inspiratory muscle function loss, which was associated with significant atrophy of the diaphragm and closely related to a reduction in PA in CF patients
**Forte *et al.* [46] 2015**	
Methods	Design: cross-sectional study Inclusion criteria: 16 years or older and confirmed diagnosis of CF Exclusion criteria: pulmonary exacerbation in the previous 30 days, cardiac or neurological disease or other chronic disease that limited the study procedures, current treatment with sedative or anti-epileptic drugs, and pregnancy
Participants	51 participants with CF 47% female, mean±sd age 25.1±8.8 years, BMI 20.5±2.4 kg·m^−2^
Intervention	Nutritional and sleep questionnaires were answered by the participants Spirometry and the 6MWT performed Echocardiography and polysomnography data also collected as part of the study
Outcomes	This study examined PASP, Epworth sleepiness scale, Pittsburgh sleep quality index score, sleep stage score, rapid eye movement, apnoea/hypopnoea index and 6MWD
Notes	A range of questionnaires, including the World Health Organization Quality of Life and the Cystic fibrosis Quality of Life, were utilised in this study
**Giannakoulakos *et al.* [75] 2022**	
Methods	Design: case–control study
Participants	45 participants with CF CF group: mean±sd age 13.22±4.6 years, BMI 19.58±4.1 kg·m^−2^ Healthy control group: mean±sd age 13.80±4.5 years, BMI 19.57±4.2 kg·m^−2^
Intervention	Subjects completed two self-administered validated questionnaires, the Godin Leisure-Time Exercise Questionnaire and the DISABKIDS for QoL The CF group performed spirometry and multiple breath washout tests In addition, weight, height and BMI were recorded The Godin Leisure-Time Exercise Questionnaire was used to evaluate PA, QoL was assessed using the DISABKIDS questionnaire and the correlation of PA with QoL was also assessed
Outcomes	This study examined QoL and PA
Notes	The Godin Leisure-Time Questionnaire was used in this study with the DISABKIDS questionnaire assessing QoL
**Grey *et al*. [137] 2015**	
Methods	Design: cross-sectional observational study Inclusion criteria: diagnosis of CF by sweat test chloride value >60 mEq·L^−1^ and a compatible clinical history, pancreatic insufficient, at least 8 years of age clinically stable for at least 3 months. Exclusion criteria: not reported
Participants	81 participants with CF 36 boys, 45 girls (n=81), mean±sd age 12.6±2.9 years, boys 12.4±2.6 years, girls 12.6± 3.1 years
Intervention	Participants completed a variety of tests including anthropometric and lung function, estimation of maturity (self-report), HAES PA questionnaire, completion of a food frequency questionnaire for rapid assessment of calcium- and vitamins D- and K-rich foods, DEXA for whole body assessment of bone mineral content, resting blood measurements of analysis of calcium and alkaline phosphatase, serum analysis of CRP, 25OHD, parathyroid hormone, vitamin K, osteocalcin (total and undercarboxylated), and carboxy-terminal PICP (a marker of bone formation), and citrated plasma for citrated plasma for protein induced by vitamin K absence or antagonist-II The patients also provided a mid-stream urine sample (first morning void) for urine calcium, creatinine and sodium
Outcomes	The primary outcomes were vitamin K and calcium status
Notes	Considering that PA is a potent determinant of bone health at this pre-pubertal and pubertal maturity status, little was mentioned about the influence of PA
**Gruber *et al*. [65] 2022**	
Methods	Design: RCT Inclusion criteria: confirmed diagnosis of CF by at least two pathologic sweat tests (sweat chloride >60 mmol·L^−1^) and/or by the presence of two CF mutations Exclusion criteria: any inadequately treated diabetes mellitus or musculoskeletal problems that did not allow continuous ET
Participants	14 participants with CF Aged mean±sd 11.3±3.3 years, numbers of males and females not reported T1 (baseline) BMI mean±sd 18.2±2.5 kg·m^−2^ T3 (6 months) BMI mean±sd 17.8±2.4 kg·m^−2^ T4 (12 months) BMI mean±sd 18.0 8±2.4 kg·m^−2^
Intervention	The CFmobil project was initiated to establish sport and exercise as additional additions of CF therapy to further improve patients’ care The exercise programme was offered to all patients and information was given during the regular hospital visits at the CF centre
Outcomes	Anthropometric, lung function, cardiorespiratory fitness through a CPET, assessment of PA through an accelerometer and actigraphy reported as METs (light (<3 METs·min^−1^·day^−1^), moderate (3.0–5.9 METs·min^−1^·day^−1^) and vigorous (≥6 METs·min^−1^·day^−1^)) SED was defined as an intensity of <1.5 METs·min^−1^·day^−1^
Notes	Of the 14 children, only six children completed the partially monitored, 12-month exercise programme
**Gruber** ***et al.* [66] 2021**	
Methods	Design: pre-experimental study one-group pre-test–post-test intervention study Inclusion criteria: aged 6–17 years, with CF and in a stable condition
Participants	31 participants with CF 18 males, mean±sd age 11.5±3.3 years
Intervention	Spirometry was carried out, followed by an incremental cycling test on a cycle ergometer HR was monitored with an HR monitor and chest strap Participants were encouraged to exert maximal effort DMT used to assess motor performance PA was assessed using an accelerometer over a 4-week period before any testing
Outcomes	This study examined DMT, steps·day^−1^, SED, light/moderate-to-vigorous/vigorous intensity activity
Notes	This study was registered at ClinicalTrials.gov (NCT03518697)
**Gruet *et al.* [47] 2016**	
Methods	Design: cross-sectional study Inclusion criteria: CF diagnosis based on clinical features and genotype Exclusion criteria: 18 years old, had unstable major nonpulmonary comorbidities, colonised with *B. cepacia*, had contraindications for exercise testing, had symptoms or signs of an acute pulmonary exacerbation, or had received a lung transplant
Participants	25 participants with CF Mean±sd age 30±9 years, 17 males, BMI 20.4±1.9 kg·m^−2^
Intervention	Habitual PA level and self-esteem data collected *via* questionnaires Exercise testing completed in a random order, comprising the 1-min sit-to-stand test, 6MWT, quadriceps force, pulmonary function and cardiopulmonary exercise tests
Outcomes	This study examined exercise testing results, PA level, physical self-perception profile, pulmonary function and CFTR genotype
Notes	This study used the Adherence to Quantitative Activity Protocol questionnaire to assess PA
**Gupta *et al.* [77] 2019**	
Methods	Design: randomised control study Inclusion criteria: confirmed CF, not having required *i.v.* antibiotics prior to 1 month of enrolment and FEV_1_ ≥20% pred Exclusion criteria: children with any prior diagnosed musculoskeletal disorder such as rheumatoid arthritis, muscular dystrophy or chronic renal failure
Participants	52 participants with CF Experimental group (n=25) mean±sd age 147.16±33.96 months, BMI 14.23±3.16 kg·m^−2^ Experimental group (n=27) mean±sd age 152.22±40.02 months, BMI 15.03±2.97 kg·m^−2^
Intervention	Maximal exercise testing done on a treadmill, with HR being monitored by a continuous ECG HAES used to assess daily PA and a QoL questionnaire completed 24-h food recall questionnaire and fasting blood samples
Outcomes	This study examined whole body and LS BMD, pulmonary function, exercise capacity, daily PA and QoL
Notes	This study was registered with Clinical Trials Registry-India (REF/2013/01/004447)
**Gur *et al.* [97] 2020**	
Methods	Design: single-centre prospective study Inclusion criteria: aged older than 7 years Exclusion criteria: symptoms consistent with a pulmonary exacerbation during the visit or during the preceding week and patients on treatment for osteoporosis
Participants	40 participants with CF Mean±sd age 18.3±8.1 years, 21 males, BMI 20.1±13.7 kg·m^−2^
Intervention	DEXA scans, spirometry measurements were performed Lung clearance index and 6MWT measurements were obtained Handgrip strength tested, QoL and food frequency questionnaires filled out, and accelerometers in the form of smart watches were worn on the nondominant hand for a period of 7 days
Outcomes	This study examined BMD and disease severity (measured as Shwachma–Kulczycki score) Secondary outcomes included: the correlation between BMD and the other variables (pancreatic sufficient *versus* pancreatic insufficient, dietary intake, PA, pulmonary function tests, handgrip strength and QoL)
Notes	This study used the Garmin Vívosmart 4 smart watch to measure PA
**Haslam *et al.* [78] 2001**	
Methods	Design: cross-sectional study Inclusion criteria: aged between 7.0 and 13.0 years, with pre-pubertal physical development, mild lung disease on the basis of physical examination, chest radiography and spirometry, and clinically stable Exclusion criteria: shown a weight change in excess of ±1.5 kg over the 2 months preceding the study, hospitalisation in the 2 months preceding the study, comorbidities likely to confound body composition measurement (for example, diabetes mellitus, clinical or biochemical evidence of liver disease, oedema), current use of drugs likely to confound body composition measurement (for example, use of corticosteroids or growth hormone for the preceding year, use of diuretics), or severe lung disease defined as FEV_1_ less than 40% predicted for age, height and gender
Participants	22 participants with CF 14 boys, mean age 10.3 (range 7.6–12.5) years, BMI mean 15.90 (range 13.58–17.66) kg·m^−2^
Intervention	3-day weighed food diary and stool collection and fasting venous blood samples collected at time of body composition DEXA scans also performed Activities were recorded and graded on their intensity over a period
Outcomes	This study examined energy intake, PA (J·day^−1^), DEXA fat-free soft tissue, height, weight and BMD
Notes	A 7-day diary and self-report questionnaire were completed within 2 weeks of body composition analysis to assess weekly EE based on type and duration of physical activities
**Hebestreit *et al.* [86] 2006**	
Methods	Design: cross-sectional study Inclusion criteria: stable patients diagnosed with CF and aged 12 years old were recruited from CF centres in Germany (Frankfurt, Hannover and Wurzburg; n=37) and Switzerland (Basel, Bern and Zurich; n=34) Exclusion criteria: those with medical problems precluding maximal exercise testing
Participants	74 participants with CF 35 males, mean±sd age 20.5±6.0 years, body fat percentage mean±sd 15.9±7.2 %
Intervention	Skin fold thickness measured and spirometry undertaken Patients undertook a Wingate test on a cycle ergometer, with work rate increased every minute by 15–20 W, depending on patient characteristics PA measured using an accelerometer for 7 days
Outcomes	This study examined *V*′_O_2___max_, daily accelerometer counts, time spent in MVPA
Notes	This study monitored PA for 7 days using the MTI/CSA 7164 accelerometer
**Hebestreit *et al.* [85] 2010**	
Methods	Design: RCT Inclusion criteria: a confirmed diagnosis of CF (*i.e.* a typical clinical picture and at least two positive sweat tests or CF-relevant mutations on both alleles of the CFTR gene), an age of 12 years or older, an FEV_1_ of 35% or higher of predicted and the ability to perform PA Exclusion criteria: non-CF-related chronic diseases and CF-related conditions posing an increased risk to the patient when exercising
Participants	23 participants with CF PA group (n=23) mean±sd age 19.5±6.4 years, 13 male Control group (n=15) mean±sd age 19.4±5.3 years, six male
Intervention	Patients given an HR monitor and were given an individualised activity plan Encouraged to also perform strength-enhancing exercises Control patients were told to keep their activity level consistent for 12 months
Outcomes	This study examined *V*′_O_2___peak_, work capacity, muscle power, PA, lung function and QoL
Notes	This study had patients in the intervention group consent to increase their sport activities by a minimum of 3660 min·week^−1^ for the first 6 months of the study
**Hebestreit *et al.* [87] 2014**	
Methods	Design: cross-sectional analysis of separate intervention studies Inclusion criteria: with CF, aged 12 years and older and with an FEV_1_ of at least 35% pred, were recruited from German CF centres in Frankfurt, Hannover and Würzburg (n=38), and from the German-speaking provinces of Switzerland (n=39)
Participants	76 participants with CF 37 males Swiss patient group (intervention group: n=23; control group: n=15) mean±sd age 21.6±5.4 years German patient group (strength training group: n=12; aerobic training group: n=17; control group: n=10) mean±sd age 19.6±6.0 years
Intervention	QoL measure *via* questionnaire, BMI and body adiposity calculated, and spirometry undertaken Participants completed a continuous incremental cycling task to volitional fatigue, with work rate increasing every minute by 15–20 W·min^−1^ 7-day PA questionnaire filled out
Outcomes	This study examined HRQoL, reported PA, height, weight, BMI, body composition (percent body fat), pulmonary function (FEV_1_), short-term muscle power, maximal aerobic work rate, *V*′_O_2___peak_ and MVPA An accelerometer was worn for a period of 7 consecutive days Patients of the intervention groups consented to add regular strenuous exercise to their baseline activities for 6 months In the Swiss study, weekly exercise was increased by 3×30 min of supervised strength training or aerobic training In the German study, patients of the intervention group agreed to add 3×60 min·week^−1^ of any sport activity to their routine activities
Notes	Data for this project was taken from two intervention studies, one of which being Hebestreit *et al.* [85]
**Hebestreit** ***et al.* [115] 2022**	
Methods	Design: parallel-arm multicentre RCT Inclusion criteria: confirmed diagnosis of CF, aged ≥12 years, FEV_1_ ≥35% pred and access to the internet Exclusion criteria: participation in another clinical trial up to 4 weeks prior to the first baseline visit, status post-lung transplantation, pregnancy/breastfeeding, inability to exercise, more than 4 h of reported vigorous PAs per week currently or up to 3 months prior to baseline measurements and not already planned within the coming 6 months, or unstable condition affecting pulmonary function or exercise participation
Participants	1117 participants with CF Intervention group (n=60) mean±sd age 25.3±11.4 years, 45% male, BMI 22.0±4.1 kg·m^−2^ Control group (n=57) mean±sd age 22.8±10.8 years, 44% male, BMI 20.8±3.5 kg·m^−2^
Intervention	Patients were seen twice within 4 weeks, then every 3 months for 1 year thereafter PA was assessed using the 7-day recall PA questionnaire and pedometer Spirometry and skinfold thickness measurement also undertaken Participants in intervention group were asked to add at least 3 h of VPA per week, with at least 30 min of strength training and 2 h of aerobic training Control groups were told to keep their PA consistent
Outcomes	This study examined change in predicted FEV_1_ from baseline, changes in PA, exercise capacity, pulmonary function, body composition, QoL, depression, anxiety and stress scales, exercise motives as well as glycaemic control and time to first exacerbation
Notes	This study was a parallel-arm RCT (Clinicaltrials.gov identifier: NCT01744561) conducted in 27 CF centres across Austria, Canada, France, Germany, Switzerland, the Netherlands, the UK and the USA
**Ionescu *et al.* [48] 2000**	
Methods	Design: case–control study Inclusion criteria: Confirmed diagnosis of CF Exclusion criteria: patients with diabetes mellitus, liver cirrhosis, chronic respiratory failure with cor pulmonale or failure to give informed consent
Participants	22 participants with CF 11 males, mean age 23.6 (95% CI 21.4–24.7) years
Intervention	Venous blood samples obtained, spirometry undertaken, 24-h urine collected and 3-day food intake diary completed Recall questionnaires also completed
Outcomes	This study examined body composition, bone metabolism, inflammatory status, clinical status and catabolic status
Notes	This study used a recall questionnaire for the month before the assessment when patients were clinically stable to measure PA
**Jantzen *et al.* [91] 2016**	
Methods	Design: case–control study Inclusion criteria: aged 3 years or older and a proven diagnosis of CF Exclusion criteria: patients with chronic diseases other than CF that may limit PA (*i.e.*, musculoskeletal problems, neurological impairment, cardiovascular or metabolic diseases) or patients that were on the waiting list for lung transplantation or had undergone transplantation
Participants	66 participants with CF CF group (n=66) mean age 11.0 (IQR 8.6–16.0) years, 57.6% male, BMI 17.1 (IQR 15.2–20.7) kg·m^−2^ Healthy group (n=65) mean age 10.0 (IQR 7.0–19.0) years, 60% male, BMI 16.9 (IQR 15.2–20.5) kg·m^−2^
Intervention	Spirometry undertaken and an accelerometer worn for nine consecutive days on right hip
Outcomes	This study examined amount of strenuous activity
Notes	This study had participants wear an accelerometer (GT1M, ActiGraph, Pensacola, FL), with a minimum of 3 days of valid recordings including 1 weekend day, and at least 10 h of valid data each day for acceptable PA assessment
**Khiroya *et al.* [21] 2015**	
Methods	Design: case–control study Inclusion criteria: written informed consent, confirmed diagnosis of CF, aged 16 years and over, chronic infection with *P. aeruginosa*, requirement for IVAT for acute pulmonary exacerbation as assessed by the direct care team at screening and a plan for patients to receive the entire course of IVAT either in hospital or in the community Exclusion criteria: pregnancy, breastfeeding, previous lung transplantation, resting transcutaneous oxygen saturations <94%, a plan to change dosage of corticosteroids during the course of antibiotics and if patients were deemed by the treating physician to be too unwell for home
Participants	45 participants with CF Hospital treatment group (n=22) median age 27.4 years, nine males, BMI 21.5 kg·m^−2^ Community treatment group (n=23) median 33.5 years, six males, BMI 22.3 kg·m^−2^
Intervention	Subjects receiving community treatment had baseline assessments performed in the clinic area immediately following the decision to start IVAT and follow-up assessments were performed when patients re-attended immediately after completing IVAT Hospitalised subjects had baseline assessments performed within 3 days of hospital admission and follow-up assessments performed within the 3 days prior to completing IVAT At baseline and follow–up visits, subjects were asked to complete the CFQ-R, the HAES and the MST An accelerometer was worn
Outcomes	This study examined activity level, energy balance, QoL, body weight and spirometry
Notes	This study used an Actigraph® GT3X activity monitor (tri-axial accelerometer), for 3 consecutive days during the first week and 3 consecutive days during the second week of IVAT
**Klijn *et al.* [79] 2004**	
Methods	Design: RCT Inclusion criteria: children aged 9–18 years with a stable clinical condition (*i.e.*, no need for oral or *i.v.* antibiotic treatment in the 3 months prior to testing), the absence of musculoskeletal disorders and an FEV_1_ >30% pred Exclusion criteria: not reported
Participants	20 participants with CF Training group (n=11) mean±sd age 13.6±1.3 years, BMI 17.2 kg·m^−2^ Control group (n=9) mean±sd age 14.2±2.1 years, BMI 18.5 kg·m^−2^
Intervention	Randomly assigned to training group or control group Training group trained twice a week for 12 weeks with each session lasting 30–45 min Control group asked not to change their daily normal activities
Outcomes	This study examined anaerobic performance, mean power, aerobic performance and QoL
Notes	The adherence was judged to be excellent with a 98% attendance level at the exercise sessions
**Mackintosh *et al.* [61] 2018**	
Methods	Design: cross-sectional study with age- and sex-matched control group Inclusion criteria: aged 6–17 years old, no increase in symptoms or weight loss 2 weeks prior to testing and stable lung function (within 10% of best in the preceding 6 months) Exclusion criteria: unstable nonpulmonary comorbidities or acute infections
Participants	18 participants with CF (10 males) confirmed by a sweat chloride >60 mmol·L^−1^ and genotyping (8 ΔF508 homozygote, 10 ΔF508 heterozygous; 4 CFRLD) Group demographics: 18 age- and sex-matched nonclinical participants recruited from local school CF group (n=18) mean±sd age 12.4±2.8 years Control group (n=18) mean±sd age 12.5±2.7 years
Intervention	Hip-mounted ActiGraph GT3X+ accelerometer (ActiGraph LLC, Pensacola, FL) worn by both groups to assess habitual PA level over 7 consecutive days
Outcomes	This study examined lung function, body stature and mass, waist circumference, SED, low LPA, high LPA, LPA, MPA and VPA
Notes	Besides summing MPA and VPA to classify MVPA, these authors also designated the remainder of the time as either low LPA (100−799 counts·min^−1^) or high LPA (800 counts·min^−1^ <4 METs)
**Mackintosh *et al.* [62] 2019**	
Methods	Design: cross-sectional study with age- and sex-matched controls Inclusion criteria: children with CF were eligible to participate if they had no increase in symptoms or weight loss 2 weeks prior to testing and had a stable lung function (defined as within 10% of their best in the previous 6 months) Exclusion criteria: unstable nonpulmonary comorbidities or acute infections
Participants	25 participants with mild-to-moderate CF, confirmed by a sweat chloride >60 mmol·L^−1^ and genotyping (11 homozygote, 14 heterozygote; 3 CFRLD; 1 CFRD), recruited from a UK CF outpatient clinic Group demographics: 25 age- and sex-matched adolescents Healthy counterparts were recruited from local schools CF group (n=21) mean±sd age 12.1±2.6 years Control group (n=22) mean±sd age 11.7±2.7 years
Intervention	PA and SED were measured using a hip-mounted ActiGraph GT3X+ accelerometer (ActiGraph LLC, Pensacola, FL) for 7 consecutive days, advised to remove it for water-based activities (*e.g.*, bathing, swimming) or contact sports
Outcomes	This study examined lung function, body stature and mass, SED, LPA, MPA and VPA
Notes	A valid day was defined as at least 9 h of wear-time Only participants with at least 3 valid days of data, irrespective of week or weekend day, were included in the analyses
**Marin *et al.* [80] 2004**	
Methods	Design: cross-sectional study with control group Inclusion criteria: CF, children younger than 15 years Exclusion criteria: not stated
Participants	Participants with CF (nine boys, six girls) Control group children matched by age, sex (except for one participant because of difficulties in obtaining the corresponding control of the same age and sex), socioeconomic status and nutrition status CF group (n=15) mean±sd age 8.2±3.2 years Control group (n=15) mean±sd age 7.9±3.2 years
Intervention	PA was assessed in the CF group using nonspecified questionnaire during both week and weekend days Energy expended per day was estimated from REE plus the estimated energy equivalents of PA
Outcomes	This study examined body stature and mass, skinfold measurements, lung function, plasma biochemical measurements, *e.g.*, albumin, phosphate, calcium, vitamin A and E, energy intake (recall method), resting metabolic rate, PA (questionnaire), body composition *via* deuterium oxide
Notes	A highly technical protocol, the deuterium oxide (^2^H_2_O) dilution method, was used to assess TB water and FFM according to the appropriate hydration indices
**McNarry *et al.* [68] 2021**	
Methods	Design: cross-sectional study with control group Inclusion criteria: children and adolescents with CF, confirmed by a sweat chloride >60 mmol·L^−1^ and genotyping Exclusion criteria: unstable nonpulmonary comorbidities or acute infections
Participants	29 participants with CF, recruited from CF outpatient clinics in the UK and Canada Control group were healthy, free from any chronic disease and group matched for age and sex CF group (n=28) mean±sd age 12.1±3.1 years Control group (n=24) mean±sd age 11.7±2.5 years
Intervention	Participants wore a hip-mounted ActiGraph GT3X+ accelerometer (ActiGraph LLC) for 7 consecutive days and instructed to only remove the monitor for water-based activities (*e.g.*, bathing and swimming)
Outcomes	This study examined body stature and mass, lung function, SED, LPA, MPA, VPA, sleep duration, wake after sleep onset, and sleep efficiency
Notes	To be included in the PA analyses, data had to be available for a minimum of 10 h·day^−1^ of wake wear-time on any 3 days and daily sleep time had to be ≥160 min·night^−1^ with greater than 90% estimated wear-time
**Moola *et al.* [67] 2017**	
Methods	Design: RCT of feasibility Inclusion criteria: children between the ages of 8 and 18 years with a positive diagnosis of CF, inclusive of sex, culture, race and socioeconomic status Exclusion criteria: transplant candidates, medically unstable children, *e.g.*, those with acute respiratory distress or infection, patients with cognitive and intellectual disabilities, and patients residing outside of Winnipeg in the surrounding regions
Participants	13 participants with CF PA group (n=7; five girls, two boys) age range 8–18 years Control group (n=6; three girls, three boys) mean age 9–15 years
Intervention	The intervention developed an 8-week family-mediated PA counselling programme called CF Chatters
Outcomes	This study examined body stature and mass, lung function, feasibility of intervention, MVPA, and a paediatric QoL questionnaire
Notes	A 6 min·day^−1^ increase in MVPA was observed in the intervention group, from baseline to week 12 The intervention group participants demonstrated a larger decrease in SED of 49 min·day^−1^ (491±54 min·day^−1^; median: 506.5 min·day^−1^) as compared with the 27-min·day^−1^ decrease in the control group (577±87 min·day^−1^; median: 561 min·day^−1^).
**Nixon *et al.* [81] 2001**	
Methods	Design: cross-sectional study with control group Inclusion criteria: aged 7–17 years Exclusion criteria: not stated
Participants	30 participants with CF were diagnosed by an abnormal sweat test and either typical pulmonary or digestive symptoms or a positive family history Healthy control group: siblings or friends of the CF patients or children of hospital employees CF group (n=30, 18 boys, 12 girls) mean±sd age 10.8±2.9 years Control group (n=30, 17 boys, 13 girls) mean±sd age 11.4±2.2 years
Intervention	Participants completed Kriska's Modifiable Activity Questionnaire Past year activity was assessed to account for changes in PA, including changes in season and illness It was administered directly to those 12 years or older and jointly to both the parent and child when younger than 12 years of age Participants were read a list of common leisure activities and asked to indicate the activities that they had engaged in at least 10 times during the past year Participants could also report activities not included on the list For each activity indicated, more detailed information on the number of times per month and the average duration of participation (recorded as hours per time) for each activity was recorded
Outcomes	This study examined body stature and mass, lung function, PA, cardiorespiratory fitness *via* cycle ergometer, and metabolic cart for determination of *V*′_O_2___peak_
Notes	Relatively healthy children with CF engage in less vigorous PAs than their healthy, non-CF counterparts, despite having good lung function and nutritional status Higher fitness levels in turn may promote participation in more vigorous activities and subsequently help to maintain aerobic fitness, which may ultimately have an impact on survival
**Orava *et al.* [51] 2018**	
Methods	Design: cross-sectional design Inclusion criteria: aged 18 years or older and diagnosed with CF by sweat chloride testing, genotyping or both Exclusion criteria: received oral or *i.v*. antibiotics because of pulmonary exacerbations for 1 month or longer before recruitment began or who had a lung transplant
Participants	22 participants with CF (10 men, 12 women) CF group (n=22) median age 33 (range 18–67) years; all were attending the outpatient CF clinic in the Toronto Adult Cystic Fibrosis Centre at St. Michael's Hospital over a 6-month period
Intervention	Participants completed the Habitual Activity Estimation Scale, the Multidimensional Fatigue Inventory–20 and the Hospital Anxiety and Depression Scale
Outcomes	This study examined body stature and mass, lung function, PA estimation and fatigue domains
Notes	The authors reported higher level of PA is associated with a lower level of general and physical fatigue when controlling for lung function and level of depression
**Paranjape *et al.* [84] 2012**	
Methods	Design: cross-sectional Inclusion criteria: confirmed diagnosis of CF, an age of 6–16 years, no concurrent *i.v.* antibiotic treatment and the ability to perform exercise Exclusion criteria: lung function test more than 10% decline in lung function (measured by FEV_1_ % pred) compared to the previous clinic visit or required treatment with oral or *i.v.* antibiotics for a pulmonary exacerbation
Participants	78 participants with CF CF group (n=78, 33 females, 45 males) median age 10 (range 6–16) years
Intervention	A 2-month exercise regimen consisting of activities chosen by the participant designed with a clinic physical therapist Participants encouraged but not required to keep an exercise log
Outcomes	This study examined body stature and mass, lung function, exercise capacity (6MWT), habitual activity estimation scale, and the revised CF QoL questionnaire
Notes	The authors reported that girls recorded lower habitual activity and had poorer lung function than boys and that 68% of girls completing the study did not improve exercise capacity over a 2-month period
**Potter *et al.* [82] 2022**	
Methods	Design: cross-sectional Inclusion criteria: clinical diagnosis of CF, aged 18 years or under Exclusion criteria: not stated
Participants	40 participants with CF Equal proportions of patients aged under 10 years old (n=20, 50%) and 10–18 years old (n=20, 50%) Mean±sd PA for participants over the age of 10 years old was 373.6±216.5 min·week^−1^ (approximately 53 min·day^−1^) Mean±sd level achieved on the A-STEP test for aerobic fitness was 10.0±2.35 and ranged between levels 7 and 14 CF group (n=40, 19 males, 21 females) mean±sd age 9.9±4.1 years
Intervention	Patients and families were invited to participate Participants completed self-report questionnaires on ACT use and those aged ≥10 years completed a PA questionnaire (Core Indicators and Measures of Youth Health Survey) and aerobic fitness test (the A-STEP test) Participants also completed a survey to explore the tolerance and acceptability of the fitness test, and the perceived accuracy of the self-reported data collection
Outcomes	This study examined feasibility, uptake rate (the percentage of patients with CF who agreed to participate, out of the total number of patients invited) and completion rate (the percentage of participants who successfully completed the ACT survey, PA survey and fitness test, out of the total number who consented to participate) Acceptability: Likert-scale items were used to explore patients’ perspectives on the proposed assessments, including the acceptability of the length of time taken to complete the aerobic fitness test and tolerance to test (“strongly disagree” to “strongly agree”), and the perceived accuracy of the self-reported ACT and PA data collection items
Notes	Completion rate for the fitness test was 55%, due to time constraints Most participants agreed (≥90%) they could accurately provide ACT and PA data, and the assessments were tolerable and acceptable
**Putman *et al.* [100] 2021**	
Methods	Design: prospective observational multiple cohort study Inclusion criteria IVA group: confirmed diagnosis of CF and at least one copy of the G551D mutation CF control: confirmed diagnosis of CF not treated with IVA Healthy control: matched by age ±2 years (and by Tanner stage in paediatric subjects), race and gender to the IVA cohort Exclusion criteria: all participants with CF included a history of solid organ transplantation, current pregnancy, *B. dolosa* infection; those for healthy volunteers included current pregnancy, a history of medication use or disorders known to affect bone metabolism
Participants	52 participants with CF IVA group (n= 26) mean±sd age 23.1±13.1 years CF control group (n=26) mean±sd age 22.8±13.1 years Healthy control group (n=26) mean±sd age 23.8±13.2 years
Intervention	All treatments, including IVA, were managed by the subjects’ pulmonologists
Outcomes	This study examined areal BMD, body composition, volumetric BMD, bone microarchitecture and bone strength
Notes	Cortical volume, area and porosity at the radius and tibia increased significantly in adults in the IVA cohort No significant differences were observed in changes in areal BMD, trabecular microarchitecture or estimated bone strength in adults or in any outcome measures in children
**Quon *et al.* [101] 2012**	
Methods	Design: cross-sectional Inclusion criteria: aged 12 years or older and had a confirmed diagnosis of CF with genetic and/or sweat chloride testing Exclusion criteria: previously undergone solid organ transplantation
Participants	30 participants with CF CF group (n=30) mean±sd age 22±7 years
Intervention	Participants were requested to wear the pedometer during all waking hours for a total of 21 days, which comprised two “well” periods, each of 7 days duration and one “ill” period of 7 days
Outcomes	This study examined an adherence rate of using pedometer, step count, step rate, CF Respiratory Symptom Diary and lung function
Notes	This study reported that pedometer-recorded step rate correlated with self-reported PA items on the CF Respiratory Symptom Diary
**Radtke *et al.* [102] 2022**	
Methods	Design: cross-sectional Inclusion criteria: patients with CF aged 12 years and older with an FEV_1_ of at least 35% pred Exclusion criteria: participated in another clinical trial up to 4 weeks prior to the first baseline visit, pregnant or breastfeeding, lung transplantation, a high level of VPA up to 3 months prior to baseline (>4 h·week^−1^ of reported VPA), an inability to comply with the intervention and an increased risk with exercise, unstable conditions with a potential strong effect on lung function such as recent changes in medication within 1 month or less prior to screening, a planned start or stop of IVA during the trial, or colonisation with *B. cenocepacia*
Participants	103 participants with CF Participants without CFRD (n=84) mean±sd age 19 (range 15–26) years Participants with CFRD (n=19) mean±sd age 24 (range 19–32) years
Intervention	Add at least 3 h·week^−1^ of VPA to their baseline activity including at least 30 min·week^−1^ of strength-building exercises and 2 h·week^−1^ of aerobic exercise
Outcomes	This study examined CPET, pulmonary function, oral glucose tolerance test and PA
Notes	This study analysed baseline data from the ACTIVATE-CF trial, an international multi-centre RCT conducted between June 2014 and March 2016
**Ruf *et al.* [106] 2010**	
Methods	Design: cross-sectional Inclusion criteria: participants aged 12 years or higher and a proven diagnosis of CF Exclusion criteria: patients with multi-resistant bacteria and acute exacerbation at the time of assessments as defined by published criteria
Participants	41 participants with CF CF group (n=41): male patients (n=18) mean age 15.9±4.5 years, female patients (n=23) mean±sd age 17.4±6.4 years
Intervention	A regular clinical visit and wore an accelerometer for 7 consecutive days
Outcomes	This study examined lung function, activity behaviour, accelerometry, questionnaires (the Habitual Activity Estimation Scale, the 7-day PA recall questionnaire and the Lipid Research Clinics questionnaire) and aerobic fitness
Notes	The 7-day PA recall questionnaire score (0.41<r<0.56) and “active” score (r=0.33) of the HAES correlated significantly with MVPA
**Savi *et al.* [30] 2013**	
Methods	Design: cross-sectional with healthy control group Inclusion criteria: clinically stable adult CF patients Exclusion criteria: unstable medical conditions that could cause or contribute to breathlessness (i.e., cardiovascular, metabolic or other respiratory diseases) or other disorders that could interfere with exercise testing, such as neuromuscular diseases or musculoskeletal problems, exacerbation in the 4 weeks prior to the study, on the waiting list for lung transplant, and lung transplantation
Participants	20 participants with CF CF group (n=20) mean±sd age 33±8 years Control group (n=11) mean±sd age 30±4 years (age-matched)
Intervention	Patients and healthy controls wore an accelerometer to assess daily habitual PA and they were studied over 5 consecutive typical days (including 2 weekdays and 2 weekend days).
Outcomes	This study examined pulmonary function, 6MWT, CPET and HAES questionnaire
Notes	None of PA categories estimated by HAES questionnaire correlated with PA categories measured by an accelerometer
**Savi *et al.* [32] 2015**	
Methods	Design: cross-sectional Inclusion criteria: ≥18 years of age and a confirmed diagnosis of CF based on genetic testing showing two CF-causing mutations and/or two documented sweet chloride values >60 mEq·L^−1^ Exclusion criteria: pulmonary exacerbation within 4 weeks of the study period, on the waiting list for lung transplantation or had undergone lung transplantation
Participants	60 participants with CF CF group (n=60) mean±sd age 33.5±10.5 years, male (n=35) mean±sd age 36.3±11.0 years, female (n=25) mean±sd age 29.6±7.6 years
Intervention	Patients wore a multi-sensor armband for at least 5 full consecutive days
Outcomes	This study examined pulmonary function, PA and body composition
Notes	PA at moderate intensity (4.8–7.2 METs) or greater (>7.2 METs) was independently associated with gender and FEV_1_ % pred (p=0.007 and p=0.04, respectively) Compared with men, women had reduced VPAs (p=0.01) and active EE (p=0.01)
**Savi *et al.* [29] 2020**	
Methods	Design: observational, single-centre, pilot study Inclusion criteria: confirmed diagnosis of CF based on either two CF-causing mutations and/or a sweat chloride concentration during two tests of >60 mmol·L^−1^, age ≥18 years; FEV_1_ ≥30% pred, internet access and routine use of either smartphones, androids, smartwatch or a Fitbit to monitor daily PA Exclusion criteria: pulmonary exacerbation within 4 weeks of the baseline study visit, long-term oxygen therapy, comorbidities that limited PA participation, participation in another clinical trial up to 4 weeks prior to the first baseline visit or pregnancy/breastfeeding
Participants	24 participants with CF CF group (n=24) mean±sd age 37.6±11.5 years
Intervention	Static task was supine lying and active tasks comprised stair-climbing, stationary cycling and walking (modified 6MWT) Participants undertook stair-climbing in an indoor stairwell (24 steps) and were instructed to descend and ascend the stairs as they would in everyday life
Outcomes	This study examined lung function, BMI and PA
Notes	Participants were allocated into one of four arms according to their device (Smartwatch, Fitbit, Android smartphones and iOS smartphones)
**Savi *et al*. [27] 2015**	
Methods	Design: prospective case–control study Inclusion criteria: adult CF patients with mild-to-moderate lung disease (FEV_1_ 50–90% pred) Exclusion criteria: unstable medical conditions that could cause or contribute to breathlessness or other disorders that could interfere with exercise testing, such as neuromuscular diseases or musculoskeletal problems, pulmonary exacerbation within 4 weeks of the study period, on the waiting list for lung transplantation or had undergone lung transplantation
Participants	30 participants with CF CF group (n=30) mean±sd age 33±8 years Control group (n=15) mean±sd age 29±5 years
Intervention	Patients and controls wore an accelerometer to assess daily habitual PA over 5 consecutive days of their normal activities
Outcomes	This study examined an incremental CPET on a cycle ergometer, using the accelerometer to assessed PA
Notes	MPA (>4.8 METs) and MVPA (>7.2 METs) was related to *V*′_O_2__ (p=0.005 and p=0.009, respectively) and work rate (p=0.004 and p=0.002, respectively) at lactic threshold MPA or greater was positively related to peak *V*′_O_2__ (p=0.005 and p=0.003, respectively)
**Savi *et al.* [28] 2018**	
Methods	Design: cross-sectional with control group Inclusion criteria: patients attending a CF outpatient clinic, ≥18 years of age, mild-to-moderate pulmonary impairment based on FEV_1_ (mild: FEV_1_ >80% pred; moderate: FEV_1_ 40–80% pred) and a confirmed diagnosis of CF based on genetic testing showing two CF-causing mutations and/or two documented sweet chloride values >60 mEq·L^−1^ Exclusion criteria: unstable medical conditions that could cause or contribute to breathlessness (*i.e.* cardiovascular, metabolic or other respiratory diseases), disorders that could interfere with exercise testing, such as neuromuscular diseases or musculoskeletal problems, a pulmonary exacerbation in the 4 weeks prior to the study, acute respiratory failure, on oxygen therapy, on the waiting list for lung transplantation or post-transplant
Participants	34 participants with CF Dynamic hyperinflation group (n=24) mean±sd age 34.2±9.0 years Nondynamic hyperinflation group (n=10) mean±sd age 30.5±6.7 years
Intervention	Used a PA monitor to assess habitual physical activities for a period of 5 days
Outcomes	This study examined CPET (*V*′_O_2__, *V*′_CO_2__, ventilatory profile, work rate, inspiratory capacity and end-expiratory lung volume) and daily PA using an accelerometer
Notes	70% of patients responded to CPET with dynamic hyperinflation Higher incidence of dynamic hyperinflation was found in CF males compared to CF females (p=0.026)
**Savi *et al.* [31] 2019**	
Methods	Design: case study
Participants	Three participants with CF Patient 1: a 30-year-old man diagnosed with CF at 3 years of age and commenced LUM/IVA Patient 2: a 36-year-old man with CF diagnosed at 3 years of age and commenced LUM/IVA Patient 3: a 60-year-old man with CF diagnosed at 3 years of age and commenced LUM/IVA
Intervention	The combination of the corrector LUM with the potentiator IVA
Outcomes	This study examined CPET and PA
Notes	This study performed incremental CPET and assessed PA pre- and post-2 years initiation of LUM–IVA
**Schneiderman-Walker *et al.* [83] 2005**	
Methods	Design: longitudinal (period of 2 years) Inclusion criteria: CF patients 7–17 years of age who had reported participating in their “typical habitual” PA Exclusion criteria: children arriving at the clinic unwell, displaying symptoms, such as an increased cough, purulent sputum, malaise, and fever, and/or inability to participate in regular habitual PA
Participants	109 patients: girls (n=56) and boys (n=53) Girls mean±sd age 12.2±2.9 years and boys mean±sd age12.4±2.7 years
Intervention	Data were scheduled for collection for all study patients at each quarterly clinic visit over the 2-year period If a patient was not well enough to participate in their regular habitual PA, their data collection was postponed until the visit that habitual PA had resumed
Outcomes	Anthropometric measures, pulmonary function testing, HAES questionnaire, activity diary and aerobic cycle ergometer test
Notes	This study used the HAES questionnaire for a typical weekday (Tuesday, Wednesday or Thursday) and one weekend day (Saturday) to assess PA Patients were also sent home from the clinic with a 3-day (2 weekdays, one Saturday) PA diary to complete
**Scully *et al.* [99] 2022**	
Methods	Design: cross-sectional analysis comparing the dietary intake, PA, and DXA body composition measures in adolescents and adults with CF and age-, race- and gender-matched healthy volunteers Inclusion criteria: participants with CF recruited from the Massachusetts General Hospital and Boston Children's Hospital Cystic Fibrosis Center, post-pubertal (Tanner stage V) participants aged 15 years and above Exclusion criteria (CF group): history of solid organ transplantation, current pregnancy and *B. dolosa* infection (due to institutional infection control issues) Exclusion criteria (healthy volunteers): current pregnancy, a history of medications or disorders known to affect bone metabolism, cumulative use of oral glucocorticoids for more than 2 months, or BMI <18.5 or >30 kg·m−2 (or <5th percentile or >95th percentile for paediatric participants) at the time of screening
Participants	38 participants with CF (mean±sd age 27.9±2.0 years; 52.6% female) and 19 healthy volunteers (mean±sd age 28.8±2.7 years; 52.6% female) Participant age range: 15–56 years, including eight adolescents with CF and four adolescent healthy volunteers aged 15–17 years
Intervention	A cross-sectional analysis comparing the dietary intake, PA and DXA body composition measures in adolescents and adults with CF and age-, race- and gender-matched healthy volunteers Investigated how body composition correlated with pulmonary status and dietary intake in participants with CF
Outcomes	Dietary intake, PA (using the Modifiable Activity Questionnaire), DXA body composition measures and pulmonary status (FEV_1_)
Notes	Participants were recruited from a single centre Nutrition data were limited to a single 24-h diet recall
**Selvadurai *et al*. [58] 2002**	
Methods	Design: RCT Inclusion criteria: children with CF, between 8–16 years, who were admitted to the Royal Alexandra Hospital for Children for the treatment of an infectious pulmonary exacerbation Exclusion criteria: children with known pulmonary hypertension or who required daytime oxygen prior to the pulmonary exacerbation which led to the hospital admission
Participants	66 children participated in the study Aerobic training group mean±sd age 13.2±2.0 years Resistance training group mean±sd age 13.1±2.1 years Control group mean±sd age 13.2±2.0 years
Intervention	Compared groups performing aerobic and resistance training with a control group of children with CF admitted to hospital with an intercurrent pulmonary exacerbation Subjects were randomised into three groups and the randomisation was performed in sets of six, using concealed information inside opaque envelopes Aerobic training group: participated in aerobic activities for five 30-min sessions for a week Resistance training group: exercised both upper and lower limbs against a nonisokinetic resistance machine Control group: received standard chest physiotherapy, but did not attend training sessions Effect of the programme was measured at the time of discharge and 1 month after discharge from the hospital
Outcomes	FEV_1_, FVC, *V*′_O_2__, QoL score, body mass, FFM and strength
Notes	Four children initially consented, but were excluded prior to randomisation due to patient and/or parental concerns about the possibility to being randomised into the control group One subject in the control group developed haemoptysis on day 9 of admission and withdrew from the study for the subsequent 2 days
**Selvadurai *et al*. [59] 2004**	
Methods	Design: cross-sectional study Inclusion criteria: children aged between 9 and 17 years that attended the CF clinic at the Children's Hospital at Westmead, Sydney, Australia Exclusion criteria: history of pulmonary exacerbation in the 3 months prior to the study
Participants	A total of 148 children (75 girls and 73 boys) with CF and matched controls Pre-pubescent group: CF (n=70) and matched controls (n=70) Pubescent group: CF (n=78) and matched controls (n=78) Pre-pubescent group: mean±sd age in CF 10.9±0.9 years and 11.0±0.8 years in controls Pubescent group: mean±sd age in CF 14.3±1.4 years and 14.2±1.3 years in controls
Intervention	The control group was matched for age (±6 months), gender and Tanner pubertal stage, and consisted of healthy children from surrounding schools All children in the study completed the modified Bouchard activity diary The activity diary comprised a record of the dominant activity of each 15-min period of the day and night Children completed the diaries for 2 weeks To obtain an objective measure of habitual activity, each child also wore a validated accelerometer Children completed a self-assessment of their pubertal stage by reviewing standardised Tanner charts
Outcomes	Activity levels (using an activity diary), nutrition, pancreatic function, measure of fitness (aerobic capacity, anaerobic power, and activity counts) and quality of well-being score
Notes	A total of 159 children with CF agreed to participate in the study, but 11 were excluded on the basis of a recent pulmonary exacerbation
**Shelley *et al*. [26] 2022**	
Methods	Design: cross-sectional Individuals with CF Inclusion criteria: male/female ≥18 years old, confirmed diagnosis of CF, clinically stable (4 weeks without infection/*i.v*.) Exclusion criteria: any non-CF conditions that may impair ability to be physically active, unable to understand or co-operate with the study protocol Non-CF control group Inclusion criteria: male/female ≥18 years old, nonsmoker Exclusion criteria: any non-CF conditions that may impair ability to be physically active, unable to understand or co-operate with the study protocol, cardiovascular and/or respiratory disease
Participants	CF group (n=31) mean±sd age 29±6 years (male) and 22±9 years (female) Non-CF group (n=31), mean±sd age 28±9 years, male:female 18:13
Intervention	Compared PA between people with CF and non-CF peers and examined associations between PA, vascular function and health outcome measure
Outcomes	QoL (CFQ-R), PA (variables assessed using accelerometery, PA questionnaire and vascular function (flow-mediated dilatation))
Notes	The novel PA assessment methods used in the current research may have limited clinical application owing to the cost of accelerometers and the level of expertise and time required for data analysis
**Simon *et al.* [57] 2018**	
Methods	Design: cross-sectional Inclusion criteria: all participants were between 10 and 19 years of age and at baseline health (*e.g.*, no illness exacerbation) at enrolment Exclusion criteria for participants with CF: diagnosis of type 1, 2 or monogenic forms of diabetes, pulmonary exacerbation requiring hospitalisation or systemic steroids in the 6 weeks prior to participation, pregnancy, night-time gastric tube feeding Exclusion criteria for the healthy control participants: known diagnosis of diabetes or pre-diabetes, pregnancy, and any chronic disease or use of medications that may affect glucose metabolism
Participants	Control: n=11, mean±sd age 13.5±2.3 years, 36% male CF: n=43, mean±sd age 13.8±2.7 years, 53% male
Intervention	Fasting labs and an oral glucose tolerance test were performed Continuous glucose monitor was worn in the home environment for 7 days while participants underwent usual activities of daily living For sleep monitoring, participants wore an ActiWatch 2 actigraphy monitor
Outcomes	Continuous glucose monitoring, actigraphy, fasting labs and oral glucose tolerance test
Notes	The participants with CF in this study had relatively good lung function (FVC and FEV_1_) Findings may be different in CF individuals with a greater degree of lung dysfunction
**Stephens *et al.* [55] 2016**	
Methods	Design: validation study Inclusion criteria: children aged 7–18 years Exclusion criteria: medication that would affect the HR response to exercise, such as beta-blockers, or if unable to ambulate or cooperate with the testing procedures
Participants	167 subjects aged 7–18 years with a diagnosis of either JA, JDM, moderate or severe HE A or B (up to 5% clotting factor), muscular dystrophinopathies (Becker, Duchenne or other IMD), CF or CHD who had undergone a heart repair (Fontan repair or Tetralogy of Fallot repair) and 29 healthy children Mean±sd ages: CF 12.8±2.9 years; CHD 13.6±3.3 years; HE 12.4±3.3 years; IMD 12±3.4 years; JDM 13.4±2.3 years; JA 12.7±2.6 years; and healthy 13.1±2.8 years
Intervention	Each 2-h ET session consisted of anthropometric measures, pulmonary function testing and three EE protocols: resting EE, activities of daily living and exercise
Outcomes	Indirect calorimetry, accelerometery and questionnaire
Notes	This study used both the Actical (Philips Respironics, Murrysville, PA) and Actigraph 7164 (ActiGraph, Pensacola, FL) accelerometers
**Tejero *et al*. [25] 2016**	
Methods	Design: cross-sectional study Inclusion criteria: over 16 years of age diagnosed with CF and attending a CF unit, exhibiting high sweat chloride levels (>60 mmol·L^−1^), characteristic clinical features of CF, and diagnoses confirmed by repeated genetic analysis Exclusion criteria: undergone lung transplantation and/or had a recent acute exacerbation requiring treatment with antibiotics (within 6 weeks prior to the study)
Participants	50 patients (23 males and 27 females) Median age 24.4 (range 16–46) years
Intervention	TB, femoral neck and LS BMD were determined by DEXA and bone metabolism markers alkaline phosphatase, pro-collagen type 1 N-terminal pro-peptide, PICP and β-CrossLaps PA monitoring was assessed for 5 consecutive days using a portable device Exercise capacity was also determined Serum 25-hydroxyvitamin D and vitamin K were also determined in all participants
Outcomes	Exercise tolerance and daily PA parameters and nutritional parameters
Notes	This study used the SenseWear armband to measure PA
**Tejero Garcia *et al.* [24] 2011**	
Methods	Design: cross-sectional study Inclusion criteria: aged ≥16 years of age with CF (two positive sweat tests, compatible clinical and confirmation genetic) Exclusion criteria: history of transplant, antiresorptive medication use in the previous 3 years or clinical instability (respiratory infection exacerbation needing *i.v*. antibiotic treatment in the 6 weeks prior to the beginning of the study)
Participants	50 CF patients ≥16 years, male sex (n=23) and female sex (n=27)
Intervention	PA was quantified with a portable motion monitor Cardiopulmonary exercise and 6MWTs were used to assess exercise capacity BMD was obtained from DEXA of the lumbar column, hip and whole body
Outcomes	Daily PA monitoring (using a portable monitor), maximal CPET (performed on a cycle ergometer), 6MWT, BMD and radiologic evaluation of the vertebral column
Notes	“To our knowledge, no previous study has analysed the level of PA of patients with CF in real time”
**Thobani *et al*. [49] 2015**	
Methods	Design: prospective cohort observational study Inclusion criteria: CF diagnosis, clinically stable and age ≥18 years Exclusion criteria: clinic visit indicated need for hospitalisation and/or acute exacerbation
Participants	35 participants were recruited for the study, out of which 27 completed the 1-year follow-up visit Mean±sd age: 32.15±12.27 years Sex: male 13 (48%), female 14 (52%) Race: White 26 (96%), Black 1 (4%)
Intervention	Assessment of PA was done in which subjects were provided with a pedometer that they were asked to wear for 3 consecutive days quarterly for 1 year Assessment of Life-Space Assessment score
Outcomes	Mobility was assessed monthly by the Life-Space Assessment questionnaire and quarterly by a pedometer Lung function was assessed by spirometry
Notes	One limitation of this study was the small sample size Additionally, the Life-Space Assessment was designed to assess habitual mobility or “life-space,” rather than vigorous exercise or habitual PA
**Tomlinson** ***et al.* [138] 2019**	
Methods	Design: cross-sectional study Inclusion criteria: ≥14 years of age and clinically stable at the time of recruitment Exclusion criteria: not stated
Participants	Nine participants with CF CF group (n=9) mean±sd age 30.9±8.7 years
Intervention	An 8-week video-call exercise intervention supervised three times per week by an exercise therapist
Outcomes	This study examined body stature and mass, lung function, LPA, MPA, VPA, SED, CF QoL questionnaire score and feasibility (demand, acceptability and implementation)
Notes	The authors noted the potential for reducing the time normally required between meeting patients in the same facility due to cross-infection risks and viewed this as an efficient use of clinical time, increasing the practical feasibility of using Skype for exercise delivery
**Troosters *et al.* [23] 2009**	
Methods	Design: cross-sectional study Inclusion criteria: diagnosed CF and free from other conditions that could interfere with the testing procedures, *e.g.* orthopaedic, cardiac or neurological conditions Exclusion criteria: exacerbation in the 6 weeks prior to the study, on the waiting list for lung transplantation or had undergone lung transplantation
Participants	64 participants with CF CF group (n=64, 35 male, 29 female) male mean±sd age 25±6 years; female mean±sd age 27±9 years Control group (n=20, 11 male, nine female) male mean±sd age 24±3 years; female mean±sd age 26±6 years
Intervention	Measurement of PA over 5 full days using accelerometry and measurement of *V*′_O_2___peak_ through an incremental ramp cycle ergometer test and maximum handgrip and quadriceps strength
Outcomes	This study examined body stature and mass, lung function, maximal pulmonary pressure, handgrip and quadriceps force, inspiratory and expiratory pressure, MPA, VPA, steps·day^–1^, and 6MWD
Notes	There was only a modest correlation between patients’ PA and fitness, which once adjusted for other covariates, *i.e.* lung function, only 16% additional variance was explained
**Valencia-Peris *et al.* [54] 2021**	
Methods	Design: cross-sectional study Inclusion criteria: outpatient diagnosed using a genetic test for CF and treated at the hospital, abnormal sweat chloride test (>60 mmol·L^−1^), boy/girl aged 6–17 years, and one or more of the following: exocrine pancreatic insufficiency, pulmonary disease symptoms or CF history in siblings or cousins Exclusion criteria: severe lung deterioration (FEV_1_ <30%), unstable clinical condition (*i.e.*, hospitalised or poor nutritional status) or any condition (*e.g.*, musculoskeletal disorder) impairing exercise testing
Participants	44 participants with CF CF group (n=44) mean±sd age 11±3.2 years Control group (n=45) mean±sd age 11.1±3.0 years
Intervention	A sports participation survey, which was obtained from questions asking for the PA/sports practised Participants were categorised according to their participation in the following variables: organised PA, swimming activities, individual sports and team sports
Outcomes	This study examined body stature and mass, lung function, type of sports participation, SED, LPA, MPA and VPA
Notes	CF patients did not differ in any level of PA or SED compared to their healthy control group Girls with CF were the least physically active of any of the groups
**Van Biervliet *et al.* [90] 2021**	
Methods	Design: prospective pre–post-intervention Inclusion criteria: CF patients entering a rehabilitation programme, above 6 years of age with a proven diagnosis of CF and in rehabilitation programme for a minimum of 3 weeks Exclusion criteria: an interruption of the programme for >2 consecutive days and the use of steroids
Participants	39 participants with CF CF group (n=34, 17 adults, 17 children) median age 18 (IQR 12–27) years
Intervention	A 3-week residential rehabilitation on nutritional status for patients with CF
Outcomes	This study examined physical stature and mass, presence of malnutrition, DEXA bone status (bone mineral content, fat mass, FFM), pulmonary function and PA through SenseWear (estimated total EE, steps, estimated METs and time >3 METs)
Notes	The authors noted that the SenseWear Pro3 armband and its measured caloric expenditure could not be used to tailor the actual energy intake for patients with CF
**Walker *et al*. [53] 2015**	
Methods	Design: cross-sectional with age- and sex-matched control group Inclusion criteria: diagnosis of CF and clinically stable Exclusion criteria: not reported
Participants	Six participants with CF (part of a study across multiple groups with different chronic diseases, *i.e.*, brain tumour, HE and type 1 diabetes mellitus) PA group (n=6) mean±sd age 15.0±2.5 years; four males two females Control group (n=6) mean±sd age not reported but stated as age- and sex-matched
Intervention	PA was measured using activity monitors but with a focus on assessing SED over 7 consecutive days Wear time had to be for at least 10 h·day^–1^ and at least 4 of 7 days (one of which being a weekend day) Types of screen-based sedentary behaviours were assessed using a questionnaire, which asked each parent to indicate the number of hours in a typical day their child spent watching television, using a computer or playing video games
Outcomes	This study examined SED in h·day^−1^, min·h^−1^ and % of wear time, also classifications of hours spent watching television, computer and video games
Notes	This study found that there was not a significant difference in SED (min·h^−1^ or % wear time) between the control group or when compared to other disease conditions
**Ward *et al.* [17] 2013**	
Methods	Design: prospective observational design Inclusion criteria: adults (aged >18 years) with CF who were admitted to the Royal Adelaide Hospital with an acute respiratory exacerbation (based on patient's symptoms and signs (*e.g.*, changes in sputum volume/colour/consistency, shortness of breath, fatigue, weight loss, deterioration in spirometry/oxygenation and radiography findings) Exclusion criteria: unwilling to participate, unable to commit to the 1-month follow-up measurements, a musculoskeletal condition adversely affecting PA or a medical condition considered terminal
Participants	24 participants with CF PA group (males n=15, nine females) mean age 28.3±8.4 years
Intervention	The SenseWear® Pro3 armband measured PA between days 3 and 5 of hospitalisation and again 1 month after hospital discharge MST and hand grip strength test were also assessed but not on the day PA was measured
Outcomes	The primary outcome was the time spent performing PA over a 24-h period represented as total EE (kilocalories), total PA duration, time lying down and sleep duration, and time spent in sedentary/moderate/vigorous/very vigorous activity based on the MET values Secondary outcomes were distance covered in metres for shuttle walk and grip strength in kilogrammes
Notes	Whilst 24 completed the study, 36 were recruited with 12 failing to complete the study including three who did not tolerate the armband
**Weibolt *et al.* [139] 2012**	
Methods	Design: prospective observational study Inclusion criteria: admitted to hospital with an acute exacerbation Exclusion criteria: any other medical conditions significantly reducing mobility or if they were not expected to be discharged
Participants	Participants with CF recruited at admission for acute acerbation and 25 patients with CF attending routine follow-up clinics and a named stable cohort recruited as a comparison Convalescent group (n=17) mean±sd age 29.8±8.6 years, four females, 13 males Stable group (n=25) mean±sd age 33.7±11.6 years, 10 females, 15 males
Intervention	Within 48 h of admission, participants had lung function and body composition collected, an isometric strength quadriceps test, blood tests, respiratory muscle strength *via* inspiratory and expiratory mouth pressures and an e-AR monitor for PA The stable group had identical measurements excluding the e-AR measurement Further measures were repeated on discharge (within 24 h) and 1 month later
Outcomes	This study examined FEV_1_/FVC, FFM, BMI, quadriceps maximal voluntary contraction, blood markers; CRP, albumin, full blood count, *P*_Imax_/*P*_Emax_, the SenseWear armband and the e-AR monitor
Notes	This study used a novel, ultra-lightweight (5.6×3.5×1.0 cm, 7.4 g), activity recognition sensor device (e-AR), worn discreetly behind the ear to measure PA The device was not worn during sleep
**Wells *et al.* [88] 2008**	
Methods	Design: prospective observational study Inclusion criteria: CF but healthy with no recent history of pulmonary exacerbation in the preceding 3 months (defined as increased cough, purulent sputum and malaise) with an FEV_1_ >60% pred Exclusion criteria: unable to participate in their habitual PA (*i.e.*, due to illness)
Participants	14 participants with CF PA group (n=14) mean±sd age 16.2±4.2 years, females (n=7) 16.9±4.2 years, males (n=7) 15.6±4.9 years
Intervention	This study compared the habitual PA scores (HAES questionnaire) to an accelerometer activity monitor and an activity diary in adolescent and adult patients with CF Participants were measured over 2 consecutive weeks Results from three instruments were compared to evaluate the validity and reliability of the HAES
Outcomes	This study examined activity time at different intensities (inactive, somewhat inactive, somewhat active, active) in hours and at various times of day
Notes	This study reported that the HAES is clinically feasible due to its ease of administration and high compliance/completion compared to pedometers and accelerometers
**Welsner *et al.* [22] 2021**	
Methods	Design: pre-experimental one-group pre-test–post-test interventional study Inclusion criteria: ≥18 years and diagnosis of CF made on the basis of the detection of two CF-defining mutations or two pathological sweat tests (sweat chloride >60 mmol·L^−1^) Exclusion criteria: cor pulmonale, decompensated heart failure, musculoskeletal disorders that do not allow continuous training or an insufficiently treated diabetes mellitus
Participants	26 participants with CF PA group (n=26) mean±sd age 26.5±7.9 years, eight females, 18 males
Intervention	The subjects participated in an individual training programme in collaboration with a sport and exercise scientist The individual exercise programme was developed considering age/gender, body functions, disease-related restrictions, personal factors (individual interests and inclinations, and environmental factors, *e.g.*, availability of appropriate training facilities at home or place of residence
Outcomes	This study examined anthropometry, lung function and physical fitness Activity was recorded at three time-points: baseline (T0), after 6 months (T1) and after 12 months (T2)
Notes	This study was registered on ClinicalTrials.gov (NCT03518697)

6MWD: 6-min walking distance; 6MWT: 6-min walking test; ACT: airway clearance technique A-STEP: Aerobic Stepping Test of Endurance and Performance; BMD: bone mineral density; BMI: body mass index; CF: cystic fibrosis; CFRD: cystic fibrosis-related diabetes; CFRLD: cystic fibrosis-related liver disease; CFTR: cystic fibrosis transmembrane conductance regulator; CHD: congenital heart disease; CPET: cardiopulmonary exercise testing; CFQ-R: Cystic Fibrosis Questionnaire Revised; CRF: cardiorespiratory fitness; CRP: C-reactive protein; DEE: diet-induced energy expenditure; DEXA: dual-energy X-ray absorptiometry; DMT: Deutscher Motorik Test; DXA: dual x-ray absorptiometry; EE: energy expenditure; ENMO: Euclidean norm minus one; ET: exercise training; FEF_25–75%_: forced expiratory flow at 25–75% of forced vital capacity; FEV_1_: forced expiratory volume in 1 s; FFM: fat-free mass; FVC: forced vital capacity; HAES: Habitual Activity Estimation scale; HE: haemophilia; HR: heart rate; HRQoL: health-related quality of life; HRV: heart rate variability; IL: interleukin; IQR: interquartile range; IMD: inherited muscle disease; IVA: ivacaftor; IVAT: *intravenous* antibiotic therapy; JA: juvenile arthritis; JDM: juvenile dermatomyositis; LPA: light physical activity; LS: lumbar spine; LUM: lumacaftor; MET: metabolic equivalent task; MPA: moderate physical activity; MST: Modified Shuttle Test; MVPA: moderate-to-vigorous physical activity; PA: physical activity; PAEE: physical activity energy expenditure; PASP: pulmonary artery systolic pressure; PEF: peak expiratory flow; *P*_Emax_: maximal expiratory pressure; *P*_Imax_: maximal inspiratory pressure; QoL: quality of life; PICP: pro-peptide type 1 procollagen; RCT: randomised control trial; REE: resting energy expenditure; RV: right ventricular; RQ: research question; SED: sedentary time; *S*_pO_2__: peripheral oxygen saturation; STROBE: Strengthening the Reporting of Observational Studies in Epidemiology; TB: total body; TEE: total energy expenditure; TNF-α: tumour necrosis factor-α; *V*′_CO_2__: carbon dioxide production; *V*′_O_2__: oxygen uptake; *V*′_O_2___max_: maximal oxygen uptake; *V*′_O_2___peak_: peak oxygen uptake; VPA: vigorous physical activity; WAT: wearable activity tracker.

There was wide heterogeneity in study designs. The majority of the studies were observational (n=57) [17, 19, 21, 23–28, 30, 32, 35, 37, 38, 40–46, 48–54, 59–63, 66, 68–73, 75, 76, 78, 80, 81, 86, 91, 93, 95–97, 99, 105, 108–111], eight were validation studies [16, 18, 19, 34, 47, 55, 88, 103], eight were intervention studies [31, 65, 77, 84, 85, 90, 94, 98], seven were feasibility/pilots [29, 33, 41, 67, 82, 89, 101], four were longitudinal studies [22, 74, 83, 87], three were randomised controlled trials [58, 79, 92] and two were case studies [56, 64]. Physical activity was the primary outcome investigated in 69 studies, with three studies identifying sleep as the primary variable [44, 46, 57]. 18 studies measured physical activity as the secondary outcome, which investigated factors associated with bone mineral density [48, 76–78, 96, 97, 100], cardiorespiratory fitness and exercise capacity [47, 66, 71, 72, 79, 102], mobility [49], body composition [48, 90, 109], CF-related diabetes (CFRD) [102], and lung function [109].

### Methodological strengths and limitations of included studies

Most included studies (71.0%; n=64) had no or minor methodological concerns, whereas 23 (25.5%) and three (3.5%) studies had moderate and serious methodological concerns, respectively (table 2). For most studies, methodological concerns were due to the poor description of recruitment and/or justification of the recruited sample size. Similarly, most studies did not align or rationalise their study to any theoretical or conceptual framework that underpinned the study. However, the most common limitation was the lack of evidence of stakeholders or/and patient and public involvement in the design or conduct of the study.

**TABLE 2:** Assessment of methodological strengths and limitations of included studies

Study, year	Theoretical or conceptual underpinning to the research	Statement of research aim(s)	Clear description of research setting and target population	Study design is appropriate to address the stated research aim(s)	Appropriate sample to address the research aim(s)	Rationale for choice of data collection tool(s)	Format and content of data collection tool is appropriate to address the stated research aim(s)	Description of data collection procedure	Recruitment data provided	Justification for analytic method selected	Method of analysis appropriate to answer the research aim(s)	Evidence that the research stakeholders have been considered in research design or conduct	Strengths and limitations critically discussed	Final rating
**Anifanti [94] 2022**	3	3	3	3	2	2	2	2	3	2	2	0	2	No/minor concerns
**Aznar [52] 2014**	2	3	3	3	2	2	3	3	2	2	3	0	0	No/minor concerns
**Béghin [108] 2003**	2	2	3	3	2	2	2	3	3	3	2	0	0	Moderate concern
**Béghin [60] 2005**	2	2	3	3	2	2	2	3	3	3	2	0	0	Moderate concern
**Bianchim [93] 2022**	3	3	3	2	2	3	3	2	1	3	3	0	3	No/minor concerns
**Bianchim [69] 2022**	2	3	3	3	2	3	3	2	1	1	2	1	2	No/minor concerns
**Boni [39] 2022**	2	2	3	3	2	2	3	2	2	2	3	0	2	No/minor concerns
**Britto [70] 2000**	2	0	1	0	1	2	2	1	0	1	1	3	0	Moderate concern
**Buntain [95] 2004**	2	3	3	3	2	3	3	3	2	3	3	3	2	No/minor concerns
**Buntain [74] 2006**	3	3	3	3	2	3	3	3	3	3	3	0	0	No/minor concerns
**Burghard [71] 2022**	3	3	3	3	2	3	3	3	3	2	3	0	3	No/minor concerns
**Burnett [40] 2020**	2	3	3	2	2	2	2	0	2	2	2	3	2	No/minor concerns
**Burtin [37] 2013**	2	3	3	3	2	3	3	3	1	1	2	0	0	No/minor concerns
**Burton [38] 2020**	3	3	3	3	2	2	3	2	2	2	3	0	3	No/minor concerns
**Campos [72] 2020**	2	3	2	3	2	2	2	2	3	2	3	2	2	No/minor concerns
**Causer [56] 2022**	3	3	3	2	0	2	3	1	1	1	0	0	1	Moderate concern
**Conway [96] 2000**	2	0	2	3	2	2	0	1	0	2	0	0	0	Serious concerns
**Cox** **[33] 2015**	3	2	2	3	0	2	3	3	0	2	3	0	2	Moderate concern
**Cox [92] 2022**	3	3	2	3	3	3	3	3	3	3	3	0	3	No/minor concerns
**Cox [110] 2019**	2	0	3	2	2	0	0	2	1	1	2	3	2	Moderate concern
**Cox [34] 2014**	3	0	1	2	0	2	2	1	1	1	2	3	3	Serious concern
**Cox [35] 2016**	1	3	3	3	3	3	3	2	0	2	3	0	2	No/minor concerns
**Curran [19] 2021**	3	2	3	3	3	3	3	3	1	2	3	0	1	No/minor concerns
**Curran [41] 2022**	1	3	2	2	2	3	3	2	2	2	2	0	2	No/minor concerns
**Curran [104]2022**	1	2	3	3	2	3	3	3	3	3	3	0	3	No/minor concerns
**Currie [42] 2017**	3	3	3	2	2	2	2	3	3	2	2	0	3	Moderate concern
**Dassios [73] 2022**	1	1	3	2	2	2	3	3	2	3	3	0	2	No/minor concerns
**De Freitas Coelho [63] 2022**	2	3	2	2	3	2	2	2	1	1	2	0	1	Moderate concern
**Decorte [43] 2017**	3	3	2	3	3	3	3	3	1	3	3	0	0	No/minor concerns
**Dhillon [18] 2018**	3	3	2	2	2	2	2	3	1	3	3	0	2	No/minor concerns
**Dietz-Terjung [105] 2021**	1	2	3	3	3	2	2	1	1	2	2	0	2	No/minor concerns
**Dobbin [44] 2005**	2	3	2	3	3	3	3	2	0	2	3	0	2	No/minor concerns
**Dwyer [16] 2009**	0	3	2	3	2	2	2	2	1	2	3	0	1	Moderate concern
**Elmesmari [64] 2022**	1	3	3	3	2	3	3	3	1	2	3	0	3	No/minor concerns
**Enright [45] 2007**	2	3	1	3	1	3	3	3	0	2	3	0	2	Moderate concern
**Forte [46] 2015**	1	3	2	3	3	3	2	2	3	2	2	0	2	No/minor concerns
**Giannakoulakos [75] 2022**	1	3	3	3	2	2	2	3	2	2	2	0	1	Moderate concern
**Grey [137] 2015**	2	3	3	3	2	3	3	3	2	2	3	0	0	Moderate concern
**Gruber** **[66] 2021**	1	3	2	2	2	2	3	2	1	1	1	0	2	No/minor concerns
**Gruber [65] 2022**	1	3	3	1	3	3	3	3	1	1	1	0	1	Moderate concern
**Gruet [47] 2016**	1	3	2	3	2	2	3	2	1	3	3	0	2	No/minor concerns
**Gupta [77] 2019**	1	2	2	3	2	1	2	3	1	1	2	0	1	Moderate concern
**Gur [97] 2020**	2	3	2	3	2	3	3	2	2	2	3	0	2	No/minor concerns
**Haslam [78] 2001**	3	3	2	3	2	2	3	2	1	1	2	0	2	No/minor concerns
**Hebestreit [86] 2006**	2	3	1	3	2	3	3	2	1	3	3	0	1	No/minor concerns
**Hebestreit [85] 2010**	2	3	3	3	3	2	3	3	3	3	3	1	2	No/minor concerns
**Hebestreit [87] 2014**	1	3	2	3	1	2	2	3	2	2	2	0	2	Moderate concern
**Hebestreit** **[98] 2022**	2	3	3	3	3	2	3	3	2	3	3	0	3	No/minor concerns
**Ionescu [48] 2000**	1	3	3	2	1	3	3	2	0	2	1	0	0	Moderate concern
**Jantzen [91] 2016**	0	3	2	2	3	3	3	3	1	2	3	0	2	No/minor concerns
**Khiroya [21] 2015**	0	3	3	2	2	2	3	3	1	2	2	0	2	Moderate concern
**Klijn [79] 2004**	1	3	3	3	3	3	3	3	2	1	2	0	0	Moderate concern
**Mackintosh [61] 2018**	1	3	3	3	1	3	3	2	0	2	3	0	2	No/minor concerns
**Mackintosh [62] 2019**	2	3	2	3	1	3	3	2	0	3	3	0	2	No/minor concerns
**Marin [80] 2004**	2	1	2	2	1	3	3	3	0	1	1	0	2	Moderate concern
**McNarry [68] 2021**	2	3	2	3	2	3	3	2	0	3	3	0	1	No/minor concerns
**Moola [67] 2017**	2	3	3	3	3	3	3	3	3	3	3	0	2	No/minor concerns
**Nixon [81] 2001**	0	3	3	2	1	3	3	3	2	1	2	0	0	No/minor concerns
**Orava [51] 2018**	3	3	2	3	2	3	2	2	1	2	3	0	2	No/minor concerns
**Paranjape [84] 2012**	0	3	3	2	1	2	2	2	1	1	2	0	3	No/minor concerns
**Potter [82] 2022**	2	3	3	3	3	3	1	3	2	3	3	2	2	No/minor concerns
**Putman [100] 2021**	0	3	3	3	1	3	3	3	1	3	2	0	3	No/minor concerns
**Quon [101] 2012**	1	3	2	3	1	1	3	2	2	1	2	0	2	No/minor concerns
**Radtke [102] 2022**	0	3	1	3	3	3	3	3	0	3	3	0	3	No/minor concerns
**Ruf [106] 2010**	0	3	3	2	2	3	3	3	0	2	2	0	2	No/minor concerns
**Savi [27] 2015**	1	3	3	3	1	2	2	2	0	2	3	0	2	No/minor concerns
**Savi [30] 2013**	0	3	2	3	2	3	3	2	0	2	2	0	1	No/minor concerns
**Savi [32] 2015**	0	3	3	3	2	3	2	3	0	2	3	0	2	No/minor concerns
**Savi [28] 2018**	0	3	3	3	3	3	2	3	0	2	3	0	1	No/minor concerns
**Savi [31] 2019**	0	1	2	2	2	2	2	2	0	2	2	0	1	No/minor concerns
**Savi [29] 2020**	1	3	3	3	2	3	2	3	2	2	2	0	2	No/minor concerns
**Schneiderman-Walker [83] 2005**	0	3	1	3	0	3	3	3	0	2	3	0	1	No/minor concerns
**Scully [99] 2022**	0	3	3	3	2	3	2	3	1	2	3	0	3	No/minor concerns
**Selvadurai [59] 2004**	2	3	3	3	3	3	2	1	2	2	2	0	1	No/minor concerns
**Selvadurai [58] 2002**	1	3	3	3	3	3	3	2	1	2	2	0	1	No/minor concerns
**Shelley [26] 2022**	1	3	3	3	1	2	3	3	3	2	3	0	3	No/minor concerns
**Simon [57] 2018**	2	3	3	2	1	3	3	3	2	2	2	0	3	No/minor concerns
**Stephens [55] 2016**	3	3	2	2	3	3	3	2	1	3	3	0	2	No/minor concerns
**Tejero [25] 2016**	1	2	2	2	2	3	3	2	0	2	2	0	1	No/minor concerns
**Tejero Garcia [24] 2011**	2	3	2	3	3	3	2	2	1	2	2	0	1	No/minor concerns
**Thobani [49] 2015**	2	3	2	3	2	3	2	2	1	1	2	0	1	Moderate concern
**Tomlinson** **[138] 2019**	2	3	2	3	1	3	2	2	1	1	2	1	1	Moderate concern
**Troosters [23] 2009**	1	3	2	3	2	3	2	3	1	3	2	0	2	No/minor concerns
**Valencia-Peris [54] 2021**	2	3	2	3	2	3	3	3	1	2	2	0	1	No/minor concerns
**Van Biervliet [90] 2021**	1	2	1	3	2	2	3	3	1	2	3	1	1	No/minor concerns
**Walker [53] 2015**	1	3	2	3	0	2	1	2	0	2	2	0	2	Moderate concerns
**Ward [17] 2013**	2	3	3	3	3	3	3	2	3	2	2	0	2	No/minor concerns
**Weibolt [139] 2012**	1	1	0	2	1	2	2	2	0	0	1	0	2	Serious concerns
**Wells [88] 2008**	0	2	0	0	2	0	1	0	1	0	0	0	3	No/minor concerns
**Welsner [22] 2021**w	0	0	0	0	3	1	0	1	3	2	1	3	1	Moderate concern

3: No/minor methodological concerns; 2: moderate methodological concerns; 1: serious methodological concerns; 0: very serious methodological concerns.

### Methods used to measure physical activity

Accelerometery was the most common method used to measure physical activity (n=57) [16–38, 41, 50, 52–56, 58–69, 72, 86–94, 103–107], with ActiGraph® (n=25) [21, 22, 26, 52–54, 57–59, 61–63, 66, 68, 69, 72, 85, 87, 88, 91–93, 103, 105, 112] and SenseWear® Pro Armband (n=20) [16, 18, 23–25, 27–33, 35–38, 50, 90, 107] being the most frequently used devices. Other accelerometers included ActivPal® (n=4) [19, 20, 41, 64], GENEActiv® (n=4) [56, 69, 89, 93], Actical® (n=1) [55] and Tritrac® (n=1) [60] and one study did not specify the accelerometer brand [67].

Questionnaires were the second most common method (n=35), with the HAES questionnaire (n=10) [30, 51, 73, 77, 79, 83, 84, 88, 103, 113] most frequently used, followed by the 7-day physical activity recall questionnaire [40, 42, 87, 96], the Modifiable Activity Questionnaire [81, 99, 100], a recall questionnaire (did not specify which one) [45, 48, 80], the Physical Activity Questionnaire for Older Children [74, 95], the Self-reported Baeke Questionnaire [43], the Transport Movements Questionnaire [60], the Use Risk Behaviour Survey [70], the International Physical Activity Questionnaire [72], the Adherence to Quantitative Activity Protocol Questionnaire [47], the Lipid Research Clinics questionnaire [103], the GODIN Physical Activity questionnaire [75], a validated habitual activity questionnaire adapted from that described by Hay *et al*. [2, 76], the Sport Participation Questionnaire [54] and the Sedentary Behaviour Questionnaire [53]. Four studies did not specify which questionnaire they used to measure physical activity [26, 71, 82, 98].

14 studies used other methods, including diaries (n=4) [58, 59, 78, 108], smartwatches (Garmin® and Fitbit®) (n=5) [19, 29, 41, 94, 97], pedometers (Omron HJ-322 U-E, Digi-Walker®) (n=4) [33, 49, 101, 102] and interviews (n=2) [33, 109].

### Reporting physical activity measurement

Studies reported physical activity using a variety of units. Most accelerometer- and questionnaire-based studies focused on outcomes such as time spent in different physical activity intensities, METs per day, time spent in sedentary, low, moderate and vigorous physical activity, and sleep time. Minutes per day of moderate-to-vigorous physical activity was most commonly reported (n=24) [17, 23, 26–28, 32, 35, 36, 51, 52, 54, 55, 61–63, 66–69, 72, 86, 93, 103, 114], followed by sedentary time (n=24) [17, 20, 26–28, 30, 32, 51–55, 61–63, 66–69, 72, 89, 93, 105, 114], low physical activity (n=21) [17, 25–28, 30, 32, 51, 52, 54, 55, 61–63, 66, 68, 69, 89, 93, 105, 114], moderate physical activity(n=18) [17, 23, 25–28, 30, 32, 51, 54, 55, 61, 62, 65, 69, 89, 103, 105] and vigorous physical activity (n=19) [17, 26–28, 30, 32, 51, 52, 54, 55, 61, 62, 66, 69, 89, 93, 103, 105, 114]. Studies using pedometers [33, 49, 101, 102] and smart watches reported daily step counts as the main outcomes [19, 29, 41, 94, 112].

Other studies used time to report physical activity, such as time spent in specific EE (n=3) [37, 64, 80], hours spent being physically active (n=10) [33, 74, 76, 77, 81, 87, 92, 95, 97, 115], days spent being physically active (n=3) [40, 70, 71], activity spent in different percentages of time (n=4) [75, 79, 83, 84] and minutes of physical activity per week (n=1) [82]. The second most common unit used to report physical activity was EE as total EE/METs (n=21) [16, 18, 23, 24, 27, 29–32, 35, 38, 42, 45, 48, 50, 60, 78, 90, 96, 108, 116], followed by number of steps (n=16) [19, 22, 23, 25, 29, 30, 41, 49, 50, 64, 90, 94, 97, 101, 102, 105]. Others calculated an index for different categories (*e.g.*, work, leisure and sport) (n=3) [43, 47, 50] or a mean score (n=3) [28, 99, 100], and two studies reported arbitrary categories (n=2) [88, 91].

10 studies measured and reported sleep; the most common unit used was total sleep duration in hours [64, 97] or minutes [27, 36, 44, 57, 68, 69, 93, 105]. Others also measured sleep onset latency (time to fall asleep) [57], wake after sleep onset (min of wake-up time after initial sleep onset) [36, 44, 57, 105], total time in bed [105] and sleep efficiency (percentage of time asleep out of total time in bed) [57, 105].

#### Collecting and processing device-based physical activity data

Most accelerometers were tri-axial (n=27) [16–21, 26, 52–54, 56, 60–66, 68, 69, 72, 89, 90, 93, 94, 104, 105], followed by bi-axial (n=15) [23–25, 27–31, 33–38, 55] and uni-axial (n=9) [49, 53, 58, 59, 86–88, 91, 103]. Devices were most commonly worn on the arm (n=20) [16–18, 23–25, 27–30, 32–38, 50, 90, 105] (two on the left side, five on the right side and seven nonspecified), followed by the waist (n=17) [21, 52–55, 60–63, 66, 68, 87, 88, 91, 97, 103, 104] (10 on the right side, one on the left side and two nonspecified), wrist (n=8) [26, 56, 57, 69, 89, 93, 94, 105], with six not specifying whether it was nondominant wrist, and mid-thigh (specifically to measure sedentary time) (n=3) [19, 20, 64]; six studies did not specify the placement [22, 59, 66, 67, 92, 108].

Regarding accelerometer data-processing, most studies used cut-points (n=15) [52–54, 56, 61, 62, 67–69, 86, 87, 89, 91, 93, 103], including Esliger *et al*. [117], Philips *et al*. [118], Freedson *et al*. [119], Evenson *et al*. [120] and Hildebrand *et al.* [121, 122], and arbitrarily absolute intensity cut-points (*e.g.* >1000 counts·min^−1^ used time spent in determined EE and MET thresholds to identify different physical activity intensities (>4.8 METS at least moderate-intensity; moderate-intensity activity >3 METs) [19, 20, 26, 54, 56, 61, 62, 68, 69, 93]. Only three studies used raw metrics such as Euclidean norm minus one and mean amplitude deviation to analyse their data [69, 93, 123].

The most common accelerometer wear duration was 7 days (n=22) [20, 23, 26, 29, 36, 50, 53–58, 61, 62, 64, 68, 69, 85, 89, 90, 93, 103], although this ranged from 1 day (n=2) [17, 60] to 1 year [94] (*i.e.* 3 days (n=1) [33], 4 days (n=3) [30, 52, 67], 5 days (n=9) [24, 25, 27, 28, 31, 32, 63, 86, 87], between 5 and 7 days (n=2) [35, 38], 9 days (n=1) [91], 12 days (n=1) [59], 14 days (n=1) [88], 28 days (n=2) [22, 65, 66, 105], up to 40 days (n=1) [37], and 12 weeks (n=1) [92]). Most studies instructed continuous wear, removing devices only for water-based activities [23, 27–29, 32, 54, 61, 62, 67–69, 88, 93, 124]. Wear-time inclusion criteria varied, with many requiring ≥3 days of ≥9–10 h·day^−1^ [33, 35, 52–54, 62, 69, 86, 89, 91, 93, 103, 104]. Some studies also specified whether measurement should include weekdays and weekends (n=10) [19, 25, 28, 31, 35, 53, 62, 69, 93, 104].

Four studies used accelerometers during structured protocols [16, 18, 19, 34], although approaches varied. Protocols included treadmill or self-paced walking [16, 19], structured physical tasks (*e.g.*, sitting, standing, cycling and stair-climbing) [34] and lifestyle-type activities (*i.e.*, walking on flat surfaces, walking on incline, sit-to-stand and lift-bend tests), followed by a sub-maximal steady-state cycling test [18].

### Health outcomes

Most studies also measured health outcomes, with lung function measured *via* spirometry being the most common. Specifically, 72 studies reported forced expiratory volume in 1 s (FEV_1_) (% predicted) [16–20, 22–30, 33, 35–38, 41–47, 49–51, 53, 57–63, 66–69, 71, 72, 74, 76, 77, 79, 81, 83–93, 95–97, 99–103, 105, 107, 114, 115], 24 reported FEV_1_ in litres [17, 18, 22, 26, 27, 30, 32, 35–37, 42, 43, 47, 50, 63, 69, 72, 75, 78, 89, 92, 93, 97, 105], 45 studies reported forced vital capacity (FVC) (% predicted) [16, 18–20, 22–30, 41, 43, 44, 46, 47, 50, 51, 57, 58, 60–63, 65, 68, 69, 72, 74, 75, 77, 85, 86, 88–90, 92, 93, 97, 102, 103, 105, 115] and five reported the FEV_1_/FVC ratio [22, 27, 28, 43, 47, 63, 72, 94]. Commonly reported demographic outcomes included CFRD (30 studies) [22, 24, 26, 27, 29, 32, 33, 35–37, 42, 47, 57, 62, 69, 71–74, 91–93, 95, 96, 98–100, 102, 105, 107, 125] and pancreatic insufficiency (25 studies) [22, 25, 27–29, 32–34, 36, 37, 42, 47, 63, 72–74, 78, 83, 91, 94, 95, 97, 99, 104, 105, 126]. Finally, included studies also measured maximum exercise capacity (24 studies) [18, 23, 25, 27, 28, 30, 31, 43, 47, 52, 58, 59, 71, 72, 77, 79, 81, 83, 85–88, 103, 115] and heart rate (seven studies) [18, 22, 47, 63, 72, 77, 94].

## Discussion

This review investigated the measurement of physical activity, sedentary time and sleep in children and adults with CF, including 90 studies, with 9955 participants, 4021 of whom had CF. While research on physical activity in CF has grown since 2015 (*i.e.*, 51 publications since), high heterogeneity amongst studies was observed. Most studies focused on the association between physical activity and lung function, such as FEV_1_ (% pred), but there were very few longitudinal studies monitoring these health outcomes. The increasing recognition of the importance of sleep has resulted in seven studies examining this in pwCF. While most included studies were assessed as no/minor methodological concerns, none included stakeholders in the research design or conduct. Future research should consider including patient and public involvement to ensure the needs and priorities of pwCF are being addressed [127–129]. We have established key considerations for clinical practice and research based on review findings and expertise from the research team (table 3).

**TABLE 3:** Key considerations for measuring and reporting physical activity in clinical practice and research

Research question	Considerations	Setting
**Devices, questionnaires, and diaries for measuring physical activity**	Clinical practice may benefit from the use of more affordable and easy-to-use devices, such as pedometers, smartwatches, diaries and questionnaires, depending on the expertise and resources available The HAES questionnaire is widely used in CF research constituting a great option for feasible use in the clinical setting	Clinical practice
**Devices, questionnaires and diaries for measuring physical activity as an outcome measure**	As stated in the 2015 position statement [1], diaries should not be a primary or secondary end-point for assessing the efficacy of physical activity interventions However, the use of diaries can be beneficial for documenting motivation and adherence to a physical activity plan when resources or expertise to use devices are limited or unavailable	Research
**Recommended outputs for reporting physical activity measurements from devices, questionnaires, and diaries**	It is recommended that time spent in different physical activity intensities, being sedentary and asleep should be reported when using device-based monitors Validated questionnaires, especially those tested in people with CF, should be used alongside devices where possible Diaries should only replace these measures as a last resort and are better suited for capturing qualitative aspects of physical activity, such as motivation and adherence	Clinical practice and research
**Important treatment effects for physical activity measurements from devices, questionnaires, and diaries**	Despite substantial evidence published since the 2015 statement, including data from healthy populations and in people with CF, it remains clear that higher physical activity levels and reduced sedentary time are beneficial Therefore, an active lifestyle should be encouraged However, current data are insufficient to support CF-specific recommendations, so general population guidelines may be used as a foundation for physical activity counselling in CF	Clinical practice and research
**Important considerations for collecting and processing device-based physical activity (and sleep) data**	Research into physical activity and sedentary time is increasingly utilising raw data to enable more complex analyses, such as machine learning and compositional analysis The use of raw data also enhances comparability between studies Future studies are therefore advised to select devices capable of collecting and extracting accelerometer raw data, *e.g.*, ActiGraph and GENEActiv	Clinical practice and research
**Measurement and processing features for device-based physical activity measurement**	We recommend that data resolution for physical activity analysis should be at least 1 s (minimum 30 Hz) to enable clinical teams to obtain a representative account of patients’ physical activity patterns Additionally, the measurement of sleep and sedentary time is advised, although further refinement of sleep detection algorithms is needed	Clinical practice

CF: cystic fibrosis; HAES: habitual activity estimation scale.

### Research question (RQ) 1: instruments (*i.e.* devices, questionnaires and diaries) for measuring physical activity and sleep in clinical practice

The choice of physical activity measurement depends on its clinical purpose. Since 2015, the cost of accelerometers, smart watches and phone apps have significantly decreased making their use more popular. This can be observed in accelerometers being the most common choice of device in research, but their use in clinical practice is limited. Other devices such as smartwatches and phone apps have made measuring physical activity more accessible but remain unadopted as part of the clinical care and management of CF. Further clinical validation of the physical activity metrics related to pwCF and their movement behaviours are still required. For example, few devices calibrate the movement behaviours in line with the disease-specific physiological consequences of CF [130, 131]. The increasing interest in the 24-h movement behaviours with information on sleep and sedentary behaviour is a new and emerging field, which has an important role to contribute to the health and well-being of pwCF. Device-based monitors are well placed to fill this gap. However, a technological skills gap exists in data analysis, which hinders ease of use in clinical settings. More work is needed to make physical activity data more user-friendly [132]. While device-based monitors have evolved, questionnaires like the HAES and 7-day recall remain valid methods for assessing activity perception. Combining these methods with devices can provide a detailed picture of physical activity. However, recommending a device-based monitor is challenging due to regulatory requirements, although some devices (*e.g.*, ActiGraph®, GENEActiv® and Garmin®) have met regulations in North America and the EU. While most algorithms for these devices are validated in healthy populations, there are some physical activity thresholds for CF available for ActiGraph and GENEActiv.

### RQ 2: instruments for measuring physical activity and sleep as an outcome measure in research

The choice of measurement should align with the research purpose and method, with properties such as calibration, validity, reliability and feasibility being critical to both clinicians and researchers. Whilst questionnaires are useful to estimate physical activity and help with a discussion about what types of physical activity are undertaken, device-worn monitors offer more scope to quantify physical activity domains, sedentary time and sleep. In quantifying the patterns, intensities, time and volume of physical activity, device-worn monitors are more likely to unravel the relationships to health status (morbidity and mortality) for pwCF. Therefore, it is recommended that device-worn monitors be promoted to better understand and provide a more comprehensive assessment of physical activity. Currently, models such as ActiGraph®*,* GENEActiv® and SenseWear® are commonly used in research, though no single device is clearly superior. However, SenseWear®, which uses skin conductance to gauge intensity and may be influenced by CF-related sweat electrolyte abnormalities, is no longer commercially available. Research efforts to standardise operating and reporting procedures in the context of clinical outcomes should be made a priority.

### RQ 3: recommended outputs for reporting physical activity and sleep measurements

To assist researchers and clinical teams, descriptions of the measurement, such as the resolution of data capture or wear time, should be explicitly described to enable replication. Devices that also measure step count are recommended for their ease of use and understanding (*i.e.* total number of steps to be achieved in 1 day). Devices that calculate EE should be used with caution as the algorithms to calculate the EE are based on small sample sizes and lack cross-validation studies in pwCF. Whilst questionnaires remain an excellent source of qualitative data, care must be taken when estimating time or EE.

### RQ 4: important treatment effects for physical activity and sleep measurements

There currently is no consensus on what constitutes an important treatment effect for change to occur in pwCF. More information is required on the volume and its components of frequency, intensity and duration to affect a clinically meaningful change. More information on how physical activity responds to periods of stability and exacerbations in relation to health status, the influence of new drug treatments and its relationship to changes in established patient-reported outcomes (*i.e.*, lung function, body composition and quality of life). Despite that, it is important to acknowledge that global guidelines on physical activity and sedentary behaviour are available for children (age 5 years and older), adolescents and adults [133].

### RQ 5: important considerations for collecting, data reduction and analysing device-based physical activity and sleep data in clinical practice and research

Key considerations for collecting device-based physical activity data include device type, placement and measurement duration. Smartwatches and pedometers, which offer visual feedback and are easy to use, suit clinical settings, while accelerometers provide more detailed data but require technical expertise [19, 29, 41]. Regarding placement, waist-worn accelerometers have slightly better performance, while wrist-worn devices were shown to increase compliance [132]. In addition, thigh-worn devices have been used to measure sedentary time. It is also relevant to consider whether the device is positioned on the dominant or nondominant side [131]. Length of measurement is relevant as it can affect the availability of data for processing. Considerations regarding the inclusion of both weekend days and weekdays, as well as ensuring at least 9–10 h of data daily, should be made [61, 62, 68, 69, 93].

Few of the included studies using accelerometers provided detailed descriptions of the processing features and type of analysis used. To improve transparency and replicability, it is essential that studies clearly report and justify accelerometer settings and signal processing decisions. For example, studies should specify the sampling frequency and epoch used, whether any filters were applied (*e.g.*, ActiGraph low-frequency filter) and what type of data was used for processing (*i.e.* count *versus* raw) [134, 135]. Finally, the data reduction and analysis methods (*e.g.*, cut-point and equations, compositional analyses, or machine learning) should be evidence-based and transparently reported. It is noteworthy that although CF-specific cut-points were developed for young people [130], to date there are still no specific cut-points available for older adults with CF.

### RQ 6: measurement and processing features for device-based physical activity measurement and sleep in clinical settings

To be acceptable in a clinical setting, all device-based instruments must meet the relevant national, regional or local regulatory health and safety requirements. In the case of device-worn accelerometers such as those worn on the wrist, hip or leg, the opportunity to calibrate the device is a necessity to ensure reliable and valid results. The presentation of the data is expressed predominantly as minutes per day and this aligns with national or international physical activity guidelines that present achieving a set number of minutes per day or across a week as the norm.

### Potential biases in the review process

We documented and justified deviations from our registered protocol. We believe this is the most comprehensive systematic review to date of physical activity in pwCF. Specifically, a lack of standardised protocols reduced the opportunity to synthesise the data objectively. Additionally, given the volume and complexity of data, synthesis was conducted collaboratively by the author team, with methodological frameworks guiding the process.

A limitation of the current data is that most studies have a cross-sectional design while assessing long-term health outcomes relative to physical activity. There are also likely to be large variations in fitness and health status, which are often not reported. This reporting is important because even between patients who have the same disease status, the relationship of physical activity with health can be affected. Future studies should adopt a standardised and function-based assessment of fitness coupled with a device-based assessment of physical activity to examine its interaction with health status. This will help clinical teams to better understand which types of patients respond better to interventions and improve the evidence base for individualising physical fitness and activity interventions [136].

### Practical applications

Currently, there are no international or national guidelines outlined for physical activity and exercise prescription in CF. The usual recommendation is for pwCF to follow their national guidelines for healthy children, young people and adults. Clinical teams are encouraged to develop service pathways to embed exercise and physical activity habits into practice. By embedding this pathway into healthcare infrastructures, it increases the chances that better health and health behaviours will track from childhood and adolescence into adulthood, thus reducing hospital admissions, future morbidity and contributing to increasing survival rates.

### Future research

This review highlights a marked rise in physical activity research in pwCF since 2015, reflecting growing interest in its role as a health indicator. However, variation in assessment methods, data processing and analysis limits the clarity of its long-term prognostic and rehabilitation value. An international, large-scale prospective study is needed to assess outcomes such as mortality, morbidity, cost-effectiveness and quality of life. Future interventions should explore the prognostic potential of physical activity to support its integration into both clinical care and daily life.

## Conclusion

This review highlights the rapid growth in physical activity research in pwCF since 2015, particularly through device-based assessments. Despite these advancements, most devices are used in research settings due to the time and expertise required for data analysis and the limited evidence linking physical activity metrics to long-term clinical outcomes. Standardisation in data collection and processing remains a key challenge, especially for populations with complex health needs. Where feasible, raw-data-sampling devices (*e.g.*, ActiGraph, GENEActiv) worn for ≥7 days at ≥30 Hz are recommended; otherwise, validated self-report tools such as the HAES can offer practical alternatives. Clinical teams have a critical role in promoting physical activity, reducing sedentary time and supporting sleep. By continuing to work together, researchers, clinicians and their support teams can help advance the cause in understanding the role of physical activity and health in the lives of pwCF.

**Questions for future research**

1. Improving tools for clinical use
  - How can more affordable and user-friendly tools (*e.g.*, devices or questionnaires) be adapted for clinical use in CF and other specific populations?
2. Better outcome measures
  - What are the most effective and reliable ways to capture motivation and adherence when devices or advanced tools are unavailable?
3. Refining reporting standards
  - How can reporting standards for all movement behaviours evolve to better capture the qualitative and quantitative aspects of these behaviours in clinical and research settings?
4. CF-specific guidelines
  - What specific physical activity recommendations can be developed for CF populations, given the current lack of tailored guidelines?
5. Advanced analytics for data processing
  - How can machine learning and raw data analytics be applied to provide more actionable insights in physical activity research?

## Supplementary material

**Please note:** supplementary material is not edited by the Editorial Office, and is uploaded as it has been supplied by the author. Supplementary file 1: Initial framework summary table ERR-0279-2024.SUPPLEMENT Supplementary file 2: List of excluded studies ERR-0279-2024.SUPPLEMENT2
