# Supplementary material for: The 2025 European Cystic Fibrosis Society position statement on physical activity assessment in cystic fibrosis
Source: Eur Respir Rev. 2025 Jul 9;34(177):240279. doi: 10.1183/16000617.0279-2024 (PMC12249234; doi:10.1183/16000617.0279-2024)
Supplement: Supplementary file 1 [file ERR-0279-2024.SUPPLEMENT.pdf]

Supplementary File 1. Initial Framework summary table.

Framework draft 1	Research	Clinical Practice
Which devices/questionnaires/diaries represent the informed choice for the measurement of physical activity		
What output(s) should be reported from device based physical activity measurement, questionnaires, and diaries?		
What is an important treatment effect for device based physical activity measurement, questionnaires, and diaries?		
What important consideration(s) should be made when collecting and processing device based physical activity data		
Specifically for device-based measurement of physical activity, what are the measurement and processing properties/features that should be standard for feasible utility in a clinical setting?		
