# Supplementary material for: The 2025 European Cystic Fibrosis Society position statement on physical activity assessment in cystic fibrosis
Source: Eur Respir Rev. 2025 Jul 9;34(177):240279. doi: 10.1183/16000617.0279-2024 (PMC12249234; doi:10.1183/16000617.0279-2024)
Supplement: Supplementary file 2 [file ERR-0279-2024.SUPPLEMENT2.pdf]

Supplementary Excluded studies from this review

Abbott et al. Health-related quality of life in adults with cystic fibrosis: the role of coping. Journal of Psychosomatic Research 2008 64(2) 149-157.

Abbott et al. Can health-related quality of life predict survival in adults with cystic fibrosis? American Journal of Respiratory & Critical Care Medicine 2009 179(1) 54-58.

Abbott et al. Longitudinal association between lung function and health-related quality of life in cystic fibrosis. Thorax 2013 68(2) 149-154.

Adair et al. Assessing the Utility of an Outpatient Exercise Program for Children With Cystic Fibrosis: A Quality Improvement Project. Frontiers in Pediatrics 2021 9(0) 734292.

Addy et al. Early multi-dimensional assessment of Parameters to assess Response to Intra-Venous Antibiotic Treatment for pulmonary Exacerbations: The PRIVATE Study. Journal of Cystic Fibrosis 2018 17(0) S29.

Addy et al. Use of the Fitbit Charge HR to monitor physical activity, sleep and heart rate during IV therapy for pulmonary exacerbations. Journal of Cystic Fibrosis 2019 18(0) S126-S127.

Alarie. Assessment of habitual physical activity should be part of the regular assessment of CF patients. Pediatric Pulmonology 2011 46(0) 134-135.

Alarie et al. Canadian national airway clearance study: How physically active are CF patients? Pediatric Pulmonology 2012 47(0) 367.

Alarie et al. Evaluation of physical activity using the habitual activity estimation scale (HAES) questionnaire in a multicenter study. Journal of Cystic Fibrosis 2013 12(0) S28.

Allison et al. Implementing a web-based resource to improve health-related quality of life in adult cystic fibrosis patients. Journal of Cystic Fibrosis 2021 20(0) S66-S67.

Andriola et al. The physical health and activity level of patients with cystic fibrosis. Pediatric Pulmonology 2019 54(0) 388.

Anonomous et al. Research and commentary. Nursing Children and Young People 2014 26(7) 13.

Antunes Sarmento et al. Habitual activity estimation in pediatric patients with cystic fibrosis. Journal of Cystic Fibrosis 2017 16(0) S128.

Aris et al. Guide to bone health and disease in cystic fibrosis. Journal of Clinical Endocrinology & Metabolism 2005 90(3) 1888-1896.

Athanazio et al. Do physically active and not physically active adult subjects with bronchiectasis have different clinical and functional characteristics? European Respiratory Journal 2013 42, P3699.

Baranova et al. Fractures, bone loss, bone turnover and vitamin D status in Russian adult patients with cystic fibrosis. Osteoporosis International 2013 24(1) S341-S342.

Battistini et al. [Respiratory physio-kinesitherapy in cystic fibrosis: the parents' viewpoint] Pediatria Medica e Chirurgica 1988 10(0) 1-14.

Benzrath et al. Reasons for non-attendance in sport/activity program (CF mobil) in cystic fibrosis. Journal of Cystic Fibrosis 2017 16(0) S61-S62.

Bhudhikanok et al. Bone acquisition and loss in children and adults with cystic fibrosis: a longitudinal study. Journal of Pediatrics 1998 133(1) 18-27.

Blomquist et al. Physical activity and self treatment in cystic fibrosis. Archives of Disease in Childhood 1986 61(4) 362-367.

Bondarenko et al. Home-based pulmonary rehabilitation: An implementation study using the re-aim framework. ERJ Open Research 2021 7(2).

Bowen et al. Development and implementation of a cf specific outpatient pulmonary rehabilitation program. Pediatric Pulmonology 2018 53(0) 341.

Bozdemir Özel et al. Energy expenditure during daily living activities and pulmonary function in cystic fibrosis. Fizyoterapi Rehabilitasyon 2018 29(2) S46.

Bradley et al. Cystic fibrosis research in allied health and nursing professions. Journal of Cystic Fibrosis 2012 11(5) 387-392.

Bramwell et al. Home treatment of patients with cystic fibrosis using the 'Intermate': the first year's experience. Journal of Advanced Nursing 1995 22(6) 1063-1067.

Braun et al. [Insights into cystic fibrosis-related bone disease] Archives de Pediatrie 2016 23(8) 857-866

Bravo et al. Bone mineral density, lung function, vitamin D and body composition in children and adolescents with cystic fibrosis: a multicenter study. Nutricion Hospitalaria 2018 35(4) 789-795.

Bredahl et al. Sitting and Watching the Others Being Active: The Experienced Difficulties in PE When Having a Disability. Adapted Physical Activity Quarterly 2013 30(1) 40-58.

Buckley et al. A multidimensional analysis of exercise capacity amongst adults with cystic fibrosis. Journal of Cystic Fibrosis 2018 17(0) S99.

Burghard et al. Cardiorespiratory fitness and physical activity levels in the current paediatric population with cystic fibrosis. Journal of Cystic Fibrosis 2020 19(0) S140.

Burghard et al. To investigate which physical factors influence the cardiorespiratory fitness in paediatric patients with cystic fibrosis who have no ventilatory limitation during exercise (ventilatory reserve ≥15%). Journal of Cystic Fibrosis 2021 20(0) S7.

Burton et al. Inflammatory markers, physical activity and exercise tolerance in the adult cystic fibrosis population. Journal of Cystic Fibrosis 2016 15(0) S15.

Button et al. Habitual physical activity in adults with cystic fibrosis compared to controls. Journal of Cystic Fibrosis 2012 11(0) S36.

Button et al. Habitual physical activity in adults with cystic fibrosis. Pediatric Pulmonology 2012 47(0) 368.

Button et al. Validation of the international physical activity questionnaire (IPAQ) in adults with cystic fibrosis. Journal of Cystic Fibrosis 2011 10(0) S65.

Button et al. Prevalence and impact of urinary incontinence in adult men with cystic fibrosis. Journal of Cystic Fibrosis 2011 10(0) S66.Blomquist et al. Physical activity and self treatment in cystic fibrosis. Archives of Disease in Childhood 1986 61(4) 362-367.

Calvert et al. 112 Measuring health-related quality of life factors at VCU CF center. Journal of Cystic Fibrosis 2021 20(0) S56-S57.

Camara et al. Specialized coaching at home and digital tools improve the level of physical activity among cystic fibrosis patients. European Respiratory Journal 2017 50 PA1998.

Camara et al. Effects of home-based adapted physical activity in patients with cystic fibrosis: An interventional study. Journal of Cystic Fibrosis 2018 17(0) S16.

Caramia et al. [Chronic pediatric pathology and sports] Pediatria Medica e Chirurgica 1990 12(4) 333-341Cassidy et al. Seattle children's hospital outpatient exercise program: Initial data. Pediatric Pulmonology 2-14 49(0) 401.

Carlsen et al. [Physical activity and respiratory tract diseases asthma and allergy] Tidsskrift for Den Norske Laegeforening 20000 120(27) 3305-3309.

Cassidy et al. Fitness outcomes of a pediatric CF clinic exercise program. Pediatric Pulmonology 2016 51(0) 406.

Cassidy et al. Correlation of depression or anxiety scores with physical conditioning and quality of life in a pediatric CF exercise program. Pediatric Pulmonology 2017 52(0) 496-497

Chini et al. Improving physical therapy participation in patients hospitalized with a cystic fibrosis pulmonary exacerbation. Pediatric Pulmonology 2014 49(0) 401-402.

Choy et al. Timed stance performances reflect differences in age, prevalence of comorbidities, medication use, fall history and activity level: early screening for balance loss is indicated. Australasian Journal on Ageing 2007 26(1) 29-34.Cox et al. Determinants of physical activity participation in adults with cystic fibrosis. Journal of Science and Medicine in Sport 2012 15(0) S79-S80.

Clarkson et al. Fatigue prevalence and associated factors in adults with cystic fibrosis. Respirology 2021 26(0) 58.

Cohen-Cymberknoh et al. Extra-pulmonary complications in cystic fibrosis. Pediatric Pulmonology 2021 56(0) S60-S61.

Coleman et al. Increasing Wellness Through Physical Activity in Children With Chronic Disease and Disability. Current Sports Medicine Reports 2018 17(12) 425-432.

Collins et al. The physical activity questionnaire as a tool to target exercise intervention in children and adolescents with cystic fibrosis. Pediatric Pulmonology 2015 50(0) 367-368.

Combined Sections Meeting of the American Physical Therapy Association. Cardiopulmonary Physical Therapy Journal 2021 32(3) xx.

Cox et al. Physical activity participation is associated with hospital days in adults with cystic fibrosis (CF). Respirology 2014 19(0) 47.

Cox et al. Physical activity participation by adults with cystic fibrosis: An observational study. Respirology 2016 21(3) 511-518.

Cox et al. Reduced physical activity participation is associated with increased need for hospitalisation in adults with cystic fibrosis. Journal of Cystic Fibrosis 2014 13(0) S19.

Crain et al. Muscle health contributes to greater quality of life in adults with cystic fibrosis. Pediatric Pulmonology -2020 55(0) 255-256.

Currie et al. Does following the Canadian diabetes association aerobic exercise guidelines for type 2 diabetes lead to better blood glucose control in adults with cystic fibrosis related diabetes? Pediatric Pulmonology 2015 50(0) 369.

Curran et al. Reliability and validity of the ActivPAL and Fitbit Charge 2 as a measure of step count in cystic fibrosis. Journal of Cystic Fibrosis 2019 18(0) S47.

Curran et al. Biopsychological, physical activity and sedentary behaviour profile of adults with cystic fibrosis in Ireland. Journal of Cystic Fibrosis 2020 19(0) S27.

Curran et al. Physical activity is associated with aerobic capacity and lung function in adults with cystic fibrosis. Journal of Cystic Fibrosis 2021 20(0) S99.

Cox et al. Habitual physical activity intensity relates to clinical exercise assessments in adults with cystic fibrosis. American Journal of Respiratory and Critical Care Medicine 2012 185, A2808.

Dannemann et al. The physiotherapeutic treatment in adult patients with cystic fibrosis – one-year follow up. Journal of Cystic Fibrosis 2020 19(0) S148.

Davies et al. Multi-professional expertise and high performance informatics infrastructure supports innovative health technology research and clinical care in cystic fibrosis: Project Fizzyo. Journal of Cystic Fibrosis 2019 18(0) S33.

De Marchis et al. Effectiveness of a psychomotor intervention in a group of paediatric patients with cystic fibrosis hospitalized for pulmonary exacerbation: A randomized controlled study. Journal of Cystic Fibrosis 2017 16(0) S28-S29.

Decramer et al. Physical activity in patients with cystic fibrosis: A new variable in the health-status equation unravelled? European Respiratory Journal 2006 28(4) 678-679.

Dediu et al. Factors influencing lung function in patients with cystic fibrosis in western Romania. Journal of Multidisciplinary Healthcare 2021 14(0) 1423-1429.

Delaney et al. Association and Dose--Response Relationship of Self-Reported Physical Activity and Disability Among Adults ≥50 Years: National Health and Nutrition Examination Survey, 2011-2016. Journal of Aging & Physical Activity 2020 28(3) 434-441.

Denford et al. Attitudes and experiences of physical activity among an international sample of people with cystic fibrosis and their support teams. Journal of Cystic Fibrosis 2019 18(0) S164.

Derella et al. Physical activity and sleep efficiency during acute pulmonary exacerbation: Does age matter? Pediatric Pulmonology 2019 54(0) 385.

Desplanche et al. A twinned remote program of supervised adapted physical activity (APA) for French & Irish people living with cystic fibrosis improves self-reported physical activity level and physical fitness. Journal of Cystic Fibrosis 2018 17(0) S68.

de Sousa et al. Obstructive sleep apnea in children and adolescents with cystic fibrosis and preserved lung function or mild impairment: a systematic review and meta-analysis of prevalence. Sleep Medicine 2021 88(0) 36-43.

Diab Caceres et al. Cystic fibrosis: Quality of life and radiological monitoring. Archivos de Bronconeumologia – 2021 57(9) 563-564.

Diamond et al. Mobile health technology to improve emergent frailty after lung transplantation. Clinical Transplantation - 2021 35(4) e14236.

Dill et al. Longitudinal trends in health-related quality of life in adults with cystic fibrosis. Chest 2013 144(3) 981-989.

Dona et al. Correlation between different tests to assess exercise capacity in patients with cystic fibrosis (CF). Pediatric Pulmonology 2011 46(0) 358.

Donadio et al. Correlation of physical fitness with peripheral muscle strength, physical activity levels and lung function in patients with cystic fibrosis. European Respiratory Journal 2018 52 P1317.

Dorenkamp et al. Synergistic Effects of Six Chronic Disease Pairs on Decreased Physical Activity: The SMILE Cohort Study. BioMed Research International 2016 0 1-11.

Douglas et al. Establishing valid wear-time of activity trackers for the assessment of daily physical activity in children and young people with CF. Pediatric Pulmonology 2019 54(0) 387.

Douglas et al. Quantifying moderate to vigorous physical activity using heart rate in children and young people with cystic fibrosis. Journal of Cystic Fibrosis 2019 18(0) S46.

Dreger et al. Health-Related Quality of Life Predicts Intention to Perform Strenuous Physical Activity in Adults with CF. Respirology 2020 25(0) 33.

Dury et al. Identifying specific needs in adult cystic fibrosis patients: a pilot study using a custom questionnaire. BMC Pulmonary Medicine 2021 21, 1-8.

Dury et al. Identifying specific needs in adult cystic fibrosis patients: a pilot study using a custom questionnaire. BMC Pulmonary Medicine 2021 21, 270.

Dwyer et al. Relationship between physical activity and long-term health outcomes in adults with cystic fibrosis. Journal of Cystic Fibrosis 2020 19(0) S27.

Ebeling et al. Peak bone mass and primary fracture prevention. Osteoporosis International 2022 32(0) S36.

Edmunds et al. Psychological needs and the prediction of exercise-related cognitions and affect among an ethnically diverse cohort of adult women. International Journal of Sport & Exercise Psychology 2010 8(4) 446-463.

Eisenstadt et al. Evaluation of aerobic exercise capacity and daily functioning of patients with cystic fibrosis. Journal of Cystic Fibrosis 2012 11(0) S107.Almajan-Guta et al. High motivation for playing sports in cystic fibrosis - What we play is life. Journal of Cystic Fibrosis 2011 10(0) S64.

Eisenstadt et al. [Exercise Capacity and Aerobic Physical Fitness Assessment among Adolescents and Adults with Cystic Fibrosis by a Questionnaire and Exercise Tests]. Harefuah -2016 155(6) 352-6, 387, 386.

Elce et al. Supervised physical exercise improves clinical, anthropometric and biochemical parameters in adult cystic fibrosis patients: A 2-year evaluation. The clinical respiratory journal2018 12(7) 2228-2234.

Erratum regarding previously published articles (International Journal of Pediatrics and Adolescent Medicine (2018) 5(2) (49–54), (S2352646718300176), (10.1016/j.ijpam.2018.02.001)). International Journal of Pediatrics and Adolescent Medicine 2020 7(4) 212.

Estevez-Gonzalez et al. Effects of a Short-Term Resistance-Training Program on Heart Rate Variability in Children With Cystic Fibrosis—A Randomized Controlled Trial. Frontiers in Physiology 2021 12, 652029.

Falskog et al. Patients want their doctors' help to increase physical activity: a cross sectional study in general practice. Scandinavian Journal of Primary Health Care 2021 39(2) 131-138.

Faricy et al. Lower aerobic capacity and physical activity in hispanics with cystic fibrosis. Pediatric Pulmonology 2017 52(0) 391-392.

Fasan et al. Understanding psychosocial parameters affecting level of physical activity in adults with cystic fibrosis. Journal of Cystic Fibrosis 2020 19(0) S158-S159.

Feeley et al. Sleep in caregivers and children with a chronic illness. Sleep 2015 38(0) A386-A387.

Fenelly et al. A review of energy expenditure and related health outcomes in cystic fibrosis. Pediatric Pulmonology 2016 51(0) 425-426.

Ferguson et al. Physical activity and attitudes towards exercise in the cystic fibrosis (CF) population - Results from a UK national survey conducted by the Cystic Fibrosis Trust. Journal of Cystic Fibrosis 2013 12(0) S102

Ferguson et al. Are cystic fibrosis patients frail? Is 50 the new 80? Journal of Cystic Fibrosis 2016 15(0) S17.

Filleul et al. Development and validation of the Cystic Fibrosis Decisional Balance for Physical Activity scale (CF-DB-PA). BMC Pulmonary Medicine 2021 21(1) xx.

Flewelling et al. Social support is associated with fewer reported symptoms and decreased treatment burden in adults with cystic fibrosis. Journal of Cystic Fibrosis 2019 18(4) 572-576.

Flume et al. JCF Year in Review. Journal of Cystic Fibrosis 2021 20(1) 1-2.

Frangolias et al. Role of exercise and nutrition status on bone mineral density in cystic fibrosis. The clinical respiratory journal 2018 12(7) 2228-2234.

Frost et al. Detecting changes in health in cystic fibrosis-a role for smartphones? Journal of Cystic Fibrosis 2017 16(0) S51.

Fuchs et al. Role of an adapted physical activities professional at a paediatric CF centre. Journal of Cystic Fibrosis 2015 14(0) S125.

Fuchs et al. An adapted physical activities professor at a pediatric CF center: A privileged interlocutor. Pediatric Pulmonology 2015 50(0) 370.

Fuchs et al. Knowledge about factors influencing physical activity in paediatric cohort of CF patients. Journal of Cystic Fibrosis 2017 16(0) S126.

Gea et al. Nutritional status and muscle dysfunction in chronic respiratory diseases: Stable phase versus acute exacerbations. Journal of Thoracic Disease 2018 10(0) S1332-S1354.

Gee et al. Quality of life in cystic fibrosis: the impact of gender, general health perceptions and disease severity. 2003 1(4) 206-213.

Gee et al. Associations between clinical variables and quality of life in adults with cystic fibrosis. 2005 1(1) 59-66.

Gilljam et al. Passive smoking in cystic fibrosis. Respiratory Medicine 1990 84(4), 289-291.

Gold et al. Correspondence between symptoms and preference-based health status measures in the STOP study. Journal of Cystic Fibrosis 2019 18(2) 251-264.

Goldbart et al. Effects of rehabilitation winter camps at the dead sea on European cystic fibrosis patients. Israel Medical Association Journal 2007 9(11) 806-809.

Gomes et al. Common content between quality of life questionnaires for children with cystic fibrosis and the International Classification of Functionality, Disability and Health. Journal of Rehabilitation Medicine 2019 51(8) 582-586.

Gonzalez et al. Assessed physical activity in Brazilian patients with cystic fibrosis and its association with exacerbations and lung function. Pediatric Pulmonology 2016 51,(0) S27.

Good et al. Assessment of gross motor skills in children with cystic fibrosis. Pediatric Pulmonology2020 55(0) 256.

Goodwin et al. Reactions to the metaphors of disability: the mediating effects of physical activity. Adapted Physical Activity Quarterly 2004 21(4) 379-398.

Gorczynski et al. The Use of Single-Case Experimental Research to Examine Physical Activity, Exercise, and Physical Fitness Interventions: A Review. Journal of Applied Sport Psychology 2013 25(1) 148-156.

Graziano et al. Efficacy of a portable oxygen concentrator in the promotion of physical activity and the quality of life in a group of patients with cystic fibrosis: pilot study. Journal of Cystic Fibrosis 2019 18(0) S165.

Graziano et al. Effectiveness of energy conservation techniques in the performance of daily life activities in a group of patients with cystic fibrosis. Italian Journal of Pediatrics2020 46(1) S1.

Gretebeck et al. Longitudinal Change in Physical Activity and Disability in Adults. American Journal of Health Behavior 2012 36(3) 385-394.

Griffiths et al. Mobile phone step-counter data does not correlate with objective measures of exercise capacity. Journal of Cystic Fibrosis 2018 17(0) S103.

Gronowitz et al. Docosahexaenoic acid is associated with endosteal circumference in long bones in young males with cystic fibrosis. British Journal of Nutrition 2008 99(1) 160-167.

Gronowitz et al. Docosahexaenoic acid is associated with endosteal circumference in long bones in young males with cystic fibrosis. British Journal of Nutrition 2008 99(1) 160-167.

Gur et al. Bone mineral density, dietary intake and physical activity in cystic fibrosis patients. Journal of Cystic Fibrosis 2020 19(0) S130.

Hagger et al. Russian and British children's physical self-perceptions and physical activity participation. Pediatric Exercise Science 1998 10(2) 137-152.

Hall et al. The effects of Nintendo Wii exercise training in adults with cystic fibrosis. Physiotherapy (United Kingdom) 2011 97(0) eS648.

Hatziagorou et al. Physical activity and quality of life among patients with cystic fibrosis. Journal of Cystic Fibrosis 2016 15(0) S14-S15.

Hatziagorou et al. Does an individualized long-term exercise training program affect the cardiorespiratory capacity and cardiac function in young patients with cystic fibrosis? European Respiratory Journal 2019 54, PA344.

Hatziagorou et al. Does an individualised exercise program improve exercise capacity among young patients with cystic fibrosis? Journal of Cystic Fibrosis 2019 18(0) S123-S124.

Haynes et al. Activity levels of adults with cystic fibrosis. Journal of Cystic Fibrosis 2010 9(0) S70.

Haynes et al. Service evaluation of a community exercise programme for people with CF. Journal of Cystic Fibrosis 2018 17(0) S101.

Hecker et al. Management of osteoporosis in adults with cystic fibrosis. Drugs 2004 64(2)133-147.

Hedborg et al. Physical activity level in thirteen children with CF, at Stockholm CF-center. Journal of Cystic Fibrosis 2013 12(0) S108.

Hellmuth et al. Respiratory physiotherapy for children and adolescents with cystic fibrosis – is there a link between adherence and clinical health status? Journal of Cystic Fibrosis 2020 19(0) S145-S146.

Hemphill et al. Use of personal fitness devices in adults with cystic fibrosis: A prospective single group study. Pediatric Pulmonology 2018 53(0) 344.

Hilton et al. The use of serum creatinine to estimate skeletal muscle mass in cystic fibrosis. Journal of Cystic Fibrosis 2015 14(0) S40.

Hulzebos et al. Measurement of physical activity in patients with cystic fibrosis: a systematic review. Expert Review of Respiratory Medicine 2013 7(6) 647-653.

Holden et al. How active are CF children? Assessing physical activity levels at annual review. Journal of Cystic Fibrosis 2013 12(0) S107.

Holden et al. Preliminary findings of a study comparing Incremental Step Test (IST) performance and physical activity levels in children with CF. Journal of Cystic Fibrosis 2013 12(0) S107.

Howard et al. The CF couch potato! Habitual physical activity in an adult CF population. Journal of Cystic Fibrosis 2014 13(0) S107.

Hubert et al. Determinants of physical activity in adults with cystic fibrosis. Pediatric Pulmonology 2016 51(0) 375-376.

Hubert et al. [Cystic Fibrosis: Care of the Lung Disease] Revue du Praticien 2015 65(8) 1095-1099.

Hubert et al. Pulmonary and extrapulmonary determinants of physical activity in adults with cystic fibrosis. Journal of Cystic Fibrosis 2-15 14(0) S98.

Ifikhar et al. Cystic fibrosis fitness during inpatient treatment. Journal of Investigative Medicine 2022 70(2) 695.

Jamil et al. Wildfires disaster guidance: Tips for staying healthy during wildfires. American Journal of Respiratory and Critical Care Medicine 2019 199(2) P3-P4.

Jantzen et al. Cystic fibrosis and physical activity: Is there a significant difference to healthy individuals? European Respiratory Journal 2014 44, S58, P1971.

Johnson et al. Physical activity and inflammation in cystic fibrosis. Pediatric Pulmonology 2012 47(0) 373-374.

Jung et al. Meta-Analysis of Physical Activity Levels in Youth With and Without Disabilities. Adapted Physical Activity Quarterly 2018 35(4) 381-402.

Junge et al. CF and sport - Encouraging the motivation by a specific sport program. Journal of Cystic Fibrosis 2010 9(0) S71.Orenstein et al. The quality of well-being in cystic fibrosis. Chest 1989 95(2), 344-347.

Junge et al. Phase angle (PA) from bioelectrical impedance analysis (BIA) in children with cystic fibrosis (CF). Journal of Cystic Fibrosis 2012 11(0) S121.

Junge et al. Exercise capacity of adolescents with cystic fibrosis related diabetes. Pediatric Pulmonology 2013 48(0) 360.

Junge et al. Individual caloric intake in cystic fibrosis (CF)-how to calculate? Journal of Cystic Fibrosis 2015 14(0) S51.

Junge et al. Caloric intake in cystic fibrosis-search for an adequate method for dietary advice. Pediatric Pulmonology 2014 49(0) 417-418.

Junge et al. Respiratory physiotherapy for children with cystic fibrosis at infancy and early childhood-an actual state-target-analysis in consideration of parental influences on therapy adherence. Journal of Cystic Fibrosis 2017 16(0) S61.

Junge et al. Physical activity in children and adolescents with cystic fibrosis – do they move enough? Journal of Cystic Fibrosis 2020 19(0) S138.

Kalamara et al. Pulmonary rehabilitation for cystic fibrosis: A narrative review of current literature. Monaldi Archives for Chest Disease 2021 91(2).

Kamil-Rosenberg et al. How Do Physical Activity and Health Vary Among Younger, Middle-Aged, and Older Adults With and Without Disability? Journal of Aging & Physical Activity 2019 27(2) 234-241.

Kapoor et al. Determining resting heart rate in children using wearable activity monitors. European Respiratory Journal 2019 54 PA342.

Karapanagiotis et al. Exercise and sport habits in children and adolescents with cystic fibrosis. European Respiratory Journal 2013 42 P3601.

Karapanagiotis et al. Quantifying weight bearing activity in children and adolescents with cystic fibrosis. Journal of Cystic Fibrosis 2014 13(0) S92.

Katsagoni et al. Association of energy intake, physical activity and sedentary behaviour with growth and pulmonary function in children with cystic fibrosis. Journal of Cystic Fibrosis 2020 19(0) S17.

Kent et al. Habitual physical activity in children with cystic fibrosis: Reliability and relationship with quality of life and lung function. Journal of Cystic Fibrosis 2011 10(0) S64.

Khiroya et al. Physical activity, energy expenditure and quality of life in CF adults receiving intravenous antibiotics at home and in hospital. Journal of Cystic Fibrosis 2013 12(0) S29.

Kijora-Jaroszewska et al. Nutritional status and physical quality of life in patients above the 15 years of age suffering from cystic fibrosis. Clinical Nutrition 2019 38(0) S222-S223.

Klupa et al. Use of sensor-augmented insulin pump in patient with diabetes and cystic fibrosis: Evidence for improvement in metabolic control. Diabetes Technology and Therapeutics 2008 10(1)46-49.

Kocaaga et al. Physical activity, functional capacity, and anaerobic power in cystic fibrosis with and without impaired glucose tolerance. European Respiratory Journal 2020 56, 258.

Kowalski et al. Validation of the Physical Activity Questionnaire for Older Children. Pediatric Exercise Science 1997 9(2) 174-186.

Kowalski et al. Convergent validity of the physical activity questionnaire for adolescents. Pediatric Exercise Science 1997 9(4) 342-352.

Kruber et al. Do intravenous antibiotic therapies increase level of physical activity in cf? Pediatric Pulmonology 2011 46(0) 355.

Kwok et al. Physical Activity Among Adolescents With Long-Term Illnesses or Disabilities in 15 European Countries. Adapted Physical Activity Quarterly 2017 34(4) 456-465.Ku et al. Parental Influence on the Physical Activity Behaviors of Young Children With Developmental Disabilities. Adapted Physical Activity Quarterly 2020 37(4) 441-460.

Lacativa et al. Osteoporosis and inflammation. Arquivos Brasileiros de Endocrinologia e Metabologia 2010 54(2) 123-132.

Lambiase et al. Physical fitness in cystic fibrosis patients: What is the relation with airway obstruction? European Respiratory Journal 2015 46 PA1309.

Lands. Infection prevention and control in cystic fibrosis: One size fits all? Paediatric Respiratory Reviews 2020 36(0) 92-93.

Lang et al. CyFiT telehealth: protocol for a randomised controlled trial of an online outpatient physiotherapy service for children with cystic fibrosis. BMC Pulmonary Medicine 2019 19(1) 21.

Lannefors et al. Results of long term optimized physiotherapy on quality of life and activity hours. Journal of Cystic Fibrosis 2016 15(0) S33.

Larsen et al. Cardiorespiratory fitness and physical activity among Norwegian adolescents with cystic fibrosis. Journal of Cystic Fibrosis 2017 16(0) S128-S129.

Lawless et al. The impact of sleep on physical activity in children with cystic fibrosis. Pediatric Pulmonology 2017 52(0) 498.

Lawless et al. Sleep in children with cystic fibrosis and their caregivers. Pediatric Pulmonology 2016 51(0) 473.

Lee et al. Comparison of three models of actigraph accelerometers during free living and controlled laboratory conditions. European Journal of Sport Science 2013 13(3) 332-339.

Mackintosh et al. Physical activity levels of children and adolescents with cystic fibrosis in Wales. Journal of Cystic Fibrosis 2015 14(0) S98.

Marker et al. Physical activity and health-related quality of life in children and adolescents: A systematic review and meta-analysis. Health Psychology 2018 37(10) 893-903.

McCormick et al. Ivacaftor improves rhinologic, psychologic, and sleep-related quality of life in G551D cystic fibrosis patients. International Forum of Allergy & Rhinology 2019 9(3) 292-297.

McIntosh et al. Patients' experience of portacaths in cystic fibrosis: questionnaire-based study. Archives of Disease in Childhood 2015 100(7) 659-661.

McMurray et al. Feasibility of the Tritrac R3D accelerometer to estimate energy expenditure in youth. Pediatric Exercise Science 2004 16(3) 219-230.

Mandrusiak et al. A novel exercise program for young people with cystic fibrosis: Moving physiotherapy forward through targeted design. Physiotherapy (United Kingdom) 2011 97(0) eS1550.

Mandal et al. A pilot study of pulmonary rehabilitation and chest physiotherapy versus chest physiotherapy alone in bronchiectasis. Respiratory Medicine 2012 106(12) 1647-1654.

Marostica et al. Evaluation of the level of daily physical activity, lung function and exercise capacity in children and adolescents with cystic fibrosis (CF) and healthy controls. Pediatric Pulmonology 2017 52(0) 391.

McDonald et al. Screening for cardiovascular risk in children with CF. Pediatric Pulmonology 2013 48(0) 403-404.

McNarry et al. Compensatory changes in physical activity and sedentary time in children and adolescents with cystic fibrosis. Journal of Cystic Fibrosis - Volume 18, Issue 0, pp. S165 - published 2019-01-01.

Mihye et al. Parents' Beliefs and Intentions Toward Supporting Physical Activity Participation for Their Children With Disabilities. Adapted Physical Activity Quarterly 2015 32(2) 93-105.

Miranda et al. Pilates in noncommunicable diseases: A systematic review of its effects. Complementary Therapies in Medicine 2018 39(0) 114-130.

Mittaine et al. Habitual physical activity evaluation in CF children: Accelerometry feasibility and comparison with questionnaires. Journal of Cystic Fibrosis 2012 11(0) S106.

Moos-Thiele et al. The German multimodal project "Fit for Life" to optimize the CF-course in children with critical trends-regarding the subject physical activity. Journal of Cystic Fibrosis 2011 10(0) S84.

Morris et al. A survey of activity levels and sedentary time in adult CF patients. Journal of Cystic Fibrosis 016 15(0) S45.

Nap-Van Der Vlist et al. The prevalence of severe fatigue in cystic fibrosis. Journal of Cystic Fibrosis 2017 16(0) S129.

Nick et al. Utilization of an "n-of-1" study design to test the effect of ivacaftor in CF patients with residual CFTR function and FEV1 ≥40% of predicted. Pediatric Pulmonology 2014 49(0) 188-189.

Niedermayr et al. Habitual physical activity, exercise capacity and clinical status of children with CF-first results of a cross sectional, multi-centre study. Pediatric Pulmonology 2013 48(0) 356.

Nigro et al. Physical Activity Regulates TNFalpha and IL-6 Expression to Counteract Inflammation in Cystic Fibrosis Patients. International Journal of Environmental Research & Public Health [Electronic Resource] 2021 18,(9) 28.

Nigro et al. Physical activity regulates tnfα and il-6 expression to counteract inflammation in cystic fibrosis patients. International Journal of Environmental Research and Public Health 2021 18(9) 4691.

Nippins et al. Utilization and impact of physical therapy implementation in the cystic fibrosis clinic. Pediatric Pulmonology 2019 54(0) 384.

O'Connor et al. Study evaluating the effect of weekends on activity and inflammatory markers in adult patients with cystic fibrosis. Journal of Cystic Fibrosis 2012 11(0) S111.

Ochman et al. Nordic Walking in Pulmonary Rehabilitation of Patients Referred for Lung Transplantation. Transplantation Proceedings 2018 50(7) 2059-2063.

O'Donohoe et al. Adherence of subjects with cystic fibrosis to their home program: a systematic review. Respiratory Care 2014 59(11) 1731-1746.

Opdekamp et al. Sleep architecture in CF patients as assessed by the Body Media's SenseWear® Armband (SWA). Journal of Cystic Fibrosis 2015 14(0) S98.

Opdekamp et al. Nocturnal cough and arousals in CF patients during periods of pulmonary exacerbation: Impact on sleep. Journal of Cystic Fibrosis 2012 11(0) S110.

Opdekamp et al. Energy expenditure during a session of physiotherapy in CF patients. Journal of Cystic Fibrosis 2012 11(0) S35.

Opdekamp et al. Effect of a bronchial drainage session during the evening on the sleep efficiency and the night coughing episodes for cystic fibrosis inpatients. Journal of Cystic Fibrosis 2016 15(0) S89.

Orava et al. Fatigue and levels of physical activity in adults with cystic fibrosis: A pilot study. Pediatric Pulmonology 2013 48(0) 362.

Orenstein et al. The quality of well-being in cystic fibrosis. Chest 1989 95(2) 344-347.

Ozalp et al. High-intensity inspiratory muscle training in bronchiectasis: A randomized controlled trial. Respirology 2019 24(3) 246-253.

Patterson et al. Can developing an active ward ethos translate into cystic fibrosis patients “stepping up” during an inpatient stay? Physiotherapy (United Kingdom) 2020 107(0) e8.

Pereira Nunes Pinto et al. Digital technology for delivering and monitoring exercise programs for people with cystic fibrosis. Cochrane Database of Systematic Reviews 2021 6.

Perez-Gisbert et al. Effects of the COVID-19 pandemic on physical activity in chronic diseases: A systematic review and meta-analysis. International Journal of Environmental Research and Public Health 2021 18(23) 12278.

Pfirrmann et al. Applicability of a Web-Based, Individualized Exercise Intervention in Patients With Liver Disease, Cystic Fibrosis, Esophageal Cancer, and Psychiatric Disorders: Process Evaluation of 4 Ongoing Clinical Trials. JMIR Research Protocols 2018 7(5) e106.

Phan et al. Feasibility and acceptability of a medication schedule mobile application as part of CF care: A pilot, real-world, mobile health study in CF clinics. Journal of Cystic Fibrosis 2021 20(0) S140.

Pociask et al. Does an inpatient goal-setting based occupational therapy program promote independence and participation in meaningful activities for adolescents with CF? Pediatric Pulmonology 2016 51(0) 404-405.

Polito et al. Adiponectin Expression Is Modulated by Long-Term Physical Activity in Adult Patients Affected by Cystic Fibrosis. Mediators of Inflammation 2019.

Porcella et al. Assessment of Lumacaftor/Ivacaftor therapy on physical activity and exercise tolerance in adults with cystic fibrosis. European Respiratory Journal 2020 56, 364.

Porcella et al. Effects of lumacaftor/ivacaftor on physical activity and exercise tolerance in cystic fibrosis: an Italian multicentre study. Journal of Cystic Fibrosis 2020 19(0) S111-S112.

Powers et al. Self-reported physical activity does not correlate with ventilation inhomogeneity. Pediatric Pulmonology 2016 51(0) 370.

Powers et al. Step it up: Higher step count is significantly correlated with better exercise capacity in individuals with cystic fibrosis. American Journal of Respiratory and Critical Care Medicine 2017 195 A6135.

Pyl et al. Daily physical activity and peripheral muscle force in adults with cystic fibrosis compared with controls. Journal of Cystic Fibrosis 2016 15(0) S45.

Pyl et al. Influencing factors of skeletal muscle weakness in adults with cystic fibrosis. Journal of Cystic Fibrosis 2015 14(0) S97.

Radtke et al. COVID-19 pandemic restrictions continuously impact on physical activity in adults with cystic fibrosis. PLoS ONE 2021 16(9) e0257852.

Ramel et al. Evaluation of a pulmonary rehabilitation program offered to adult cystic fibrosis patients by the French cystic fibrosis centre of Roscoff. Journal of Cystic Fibrosis 2019 18(0) S125.

Rand et al Hacka Health 4 CF: the next chapter using co-design and partnerships. Journal of Cystic Fibrosis 2020 19(0) S142.

Rand et al. Development of Hacka Health 4 CF: A physiotherapy technology solution to support young people with cystic fibrosis. Journal of Cystic Fibrosis 2018 17(0) S100.

Raywood et al. Technical support requirements for remote monitoring of physiotherapy in children with cystic fibrosis. Journal of Cystic Fibrosis 2019 18(0) S158.

Richards et al. Energy cost of physical activity in cystic fibrosis. European Journal of Clinical Nutrition 2001 55(8) 690-697.

Roda et al. Importance of bone density evaluation in paediatric patients with cystic fibrosis. Journal of Cystic Fibrosis 2019 18(0) S21.

Rovedder et al. The level of physical activity, lung function and exercise capacity of children and adolescents with cystic fibrosis compared to healthy controls. Journal of Cystic Fibrosis 2020 19(0) S138.

Sahlberg et al. Physical activity, lung function and lung clearance index in children with cystic fibrosis. Journal of Cystic Fibrosis 2014 13(0) S92.

Savi et al. Habitual physical activity in cystic fibrosis. European Respiratory Journal 2012 40, P1455.

Savi et al. Assessment of daily physical activity in patients with cystic fibrosis. American Journal of Respiratory and Critical Care Medicine 2012, A2816.

Savi et al. Intensity of daily activity may affect exercise capacity and peripheral muscle function in adults with cystic fibrosis. Journal of Cystic Fibrosis 2013 12(0) S39.

Savi et al. The relationship between daily physical activity and exercise performance in adults with cystic fibrosis. European Respiratory Journal 2014 44 P1213.

Savi et al. The role of daily physical activity on exercise performance in adults with cystic fibrosis. Journal of Cystic Fibrosis 2014 13(0) S20.

Savi et al. Daily physical activity may affect aerobic exercise capacity and peripheral muscle function in adults with cystic fibrosis. American Journal of Respiratory and Critical Care Medicine 2014 189, A2856.

Savi et al. Dynamic hyperinflation and daily physical activity in adults with cystic fibrosis. European Respiratory Journal 2017 50 PA1833.

Savi et al. Physical activity measures in cystic fibrosis: could we use new electronic devices? Journal of Cystic Fibrosis 2019 18(0) S123.

Savi et al. Different methods of measuring physical activity in cystic fibrosis: Accelerometer versus new electronic devices. European Respiratory Journal 2019 54(0) pA341.

Sawyer et al. Health-related quality of life of children and adolescents with chronic illness--a two year prospective study. 2004 1(7) 1309-19.

Sawyer et al. Exercise testing and exercise training within cystic fibrosis centres across Australia and New Zealand: what is considered important and what is current practice? Internal Medicine Journal 2020 50(9) 1091-1099.

Sawyer et al. Exercise testing and exercise training within cystic fibrosis centres across Australia and New Zealand: what is considered important and what is current practice? Internal Medicine Journal2020 50(9) 1091-1099.

Schneiderman-Walker et al. Sex differences in habitual physical activity and lung function decline in children with cystic fibrosis. Journal of Pediatrics 2005 147(3) 321-326.

Schneiderman et al. Physical activity and lung health in patients with cystic fibrosis: A 7-year study. Pediatric Pulmonology 2010 45(0) 381-382.

Schneiderman et al. Physical activity and lung health in patients with cystic fibrosis: A 7-year study. Pediatric Pulmonology 2010 45(0) 381-382.Slater et al. Improving the specificity of the [13C]mixed triacylglycerol breath test by estimating carbon dioxide production from heart rate. European Journal of Clinical Nutrition 2006 60(11) 1245-1252.

Scichilone et al. Circadian rhythm of COPD symptoms in clinically based phenotypes. Results from the STORICO Italian observational study. BMC Pulmonary Medicine 2019 19 (171).

Seborga et al. Validation of two physical activity fatigue questionnaires in cystic fibrosis children. European Respiratory Journal 2011 38(S55)289.

Sehgal et al. Activity monitors in pulmonary disease. Respiratory Medicine 2019 151(0) 81-95.

Sermet-Gaudelus et al. Update on cystic fibrosis-related bone disease: a special focus on children. Paediatric Respiratory Reviews 2009 10(3) 134-142.

Shelley et al. A formative study exploring perceptions of physical activity and physical activity monitoring among children and young people with cystic fibrosis and health care professionals. BMC Pediatrics 2018 18(1) 335.

Shelley et al. Physical activity intensity profile in cystic fibrosis. Journal of Cystic Fibrosis 2019 18(0) S45-S46.

Siebert et al. Parental Influence on Physical Activity of Children with Disabilities. International Journal of Disability, Development & Education -2017 64(4) 378-390Seymour et al. Habitual physical activity self-report and perceived quality of life in a pediatric CF exercise program. Pediatric Pulmonology 2018 53(0) 372-373.

Simmich et al. Active Video Games for Rehabilitation in Respiratory Conditions: Systematic Review and Meta-Analysis. JMIR Serious Games 2019 7(1) e10116.

Singh et al. Exercise for Healthy Lungs. Indian Journal of Pediatrics 2019 86(11) 977-978.

Standal et al. Peers as resources for learning: a situated learning approach to adapted physical activity in rehabilitation. Adapted Physical Activity Quarterly 2008 25(3) 208-227.

Stephens et al. Validity of the Stage of Exercise Scale in Children with Rheumatologic Conditions. Journal of Rheumatology 2016 43(12) 2189-2198.

Spicher et al. Assessment of total energy expenditure in free-living patients with cystic fibrosis. Journal of Pediatrics 1991 118(6), 865-872.

Sputael et al. "Just move it … move it": a multidisciplinary motivational approach to improve the physical activity in children with cystic fibrosis. Journal of Cystic Fibrosis 2020 19(0) S138.

Stanghelle et al. Pulmonary function and oxygen uptake during exercise in 11-year-old patients with cystic fibrosis. Acta Paediatrica Scandinavica, 1986 75 (4), 657-661.

Strang et al. Sleep habits and patterns among youth with CF in the modulator era: A preliminary, observational study. Pediatric Pulmonology 2020 55(0) 271-272.

Stössel et al. Effects of combined exercise training during hospitalization in patients with cystic fibrosis. Journal of Cystic Fibrosis 2016 15(0) S61-S62.

Swisher et al. Perceptions of physical activity in a group of adolescents with cystic fibrosis. Cardiopulmonary Physical Therapy Journal 2008 19(4) 107-113.

Taylor et al. Coping and quality of life in patients awaiting lung transplantation. Journal of Psychosomatic Research 2008 65(1) 71-79.

Taylor et al. Efficacy of unsupervised exercise in adults with obstructive lung disease: a systematic review and meta-analysis. Thorax 2021 76(6) 591-600.

Thobani et al. Life space score and vitamin D status are associated with mobility in the adult CF population. Pediatric Pulmonology 2013 48(0) 364.

Thobani et al. Higher mobility scores in patients with cystic fibrosis are associated with better lung function. Pulmonary Medicine 2015 0 423219.

Timmons et al. Is physical activity an anti-inflammatory therapy for children with a chronic inflammatory disease? Pain Research and Management 2013 18(2) e5.

Tomaszek et al. Evaluation of selected insomnia predictors in adolescents and young adults with cystic fibrosis. 2018 1(3) 212-218.

Tomezsko et al. Energy expenditure and genotype of children with cystic fibrosis. Pediatric Research 1994 35(4) 451-460.

Tomezzoli et al. Skeletal muscle strength and its associated variables in cystic fibrosis patients with mild/moderate lung disease. Pediatric Pulmonology 2011 46(0) 358.

Tomlinson et al. Feasibility of using online video calling to engage patients in the management of cystic fibrosis. Journal of Cystic Fibrosis 2018 17(0) S102-S103.

Tucker et al. Implementing Follow-Along Physical Activity Videos with People Living With Chronic Conditions: A Feasibility Study. Worldviews on Evidence-Based Nursing 2019 16(5) 352-361.

Tzavelis et al. A soft, flexible, wearable device for cough detection in pediatric cystic fibrosis patients. Journal of Cystic Fibrosis 2021 20(0) S252.

Uluer et al. Fashion sense: What to wear to track physical activity. Pediatric Pulmonology 2019 54(0) 143.

Vandenoetelaer et al. Impact of physical activity on illness perception, self-esteem and HRQoL in children with CF. Journal of Cystic Fibrosis 2015 14(0) S130.

Van De Weert-Van Leeuwen. Regular physical activity and clinical outcomes: A real world example. Pediatric Pulmonology 2010 45(0) 195-197.

Van De Weert-Van Leeuwen. Regular physical activity and clinical outcomes: A real world example. Pediatric Pulmonology 2010 45(0) 195-197.

Vendrusculo et al. Sleep disorders, exercise capacity and daily physical activity levels in children and adolescents with cystic fibrosis. Journal of Cystic Fibrosis 2020 19(0) S37-S38.

Waller et al. A service evaluation of the feasibility and outcomes of a mobile phone monitored exercise programme for adults with cystic fibrosis. Pediatric Pulmonology 2019 54(0) 386.

Waller et al. An interim service evaluation of APP-based individual exercise programmes which are remotely monitored and progressed by a clinical physiologist specialising in exercise from a large Adult Cystic Fibrosis Centre. Journal of Cystic Fibrosis 2018 17(0) S102.

Walsh et al. Predictors of physical activity in a pre-lung transplant population. Journal of Heart and Lung Transplantation - 2014 33(4) S302.

Walsh et al. Physical activity level-a new marker of frailty in lung transplantation. Respirology 2015 20(0) 20.

Walsh et al. Low levels of physical activity predict worse survival to lung transplantation and poor early post-operative outcomes. Journal of Heart and Lung Transplantation 2016 35(8) 1041-1043.

Walters et al. The role of activity restriction in the association between pain and depression: a study of pediatric patients with chronic pain. Children's Health Care 19999 28(1) 33-50.

Ward et al. Exercise is commonly used as a substitute for traditional airway clearance techniques by adults with cystic fibrosis in Australia: a survey. Journal of Physiotherapy 2019 65(1) 43-50.

Welsner et al. Changes in actigraphy-measured habitual activity and sleep parameters during a partially supervised exercise program for adult CF patients. Pediatric Pulmonology 2018 53(0) 344.

White et al. Reliability and Validity of Physical Activity Instruments Used in Children and Youth With Physical Disabilities: A Systematic Review. Pediatric Exercise Science 2016 28(2) 240-263.

Wickerson et al. Physical activity in lung transplant candidates. Canadian Respiratory Journal 2011 18(0) 18A.

Wilson et al. Applying the Transtheoretical Model to Physical Activity Behavior in Individuals With Non-Cystic Fibrosis Bronchiectasis. Respiratory Care 2016 61(1) 68-77.

Wickerson et al. Early functional changes following hospitalization for lung transplantation. American Journal of Respiratory and Critical Care Medicine 2012, A5317.

Wu. The ins and outs of preparing CF patients for lung transplantation through rehabilitation. Pediatric Pulmonology 2019 54(0) 146-147.

Xia et al. Identifying Children with Medically Necessary Physical Activity Restrictions: Optimizing their Safe and Successful Participation with Peers and in Community...2016 North American Society for Pediatric Exercise Medicine (NASPEM) Biennial Meeting, Knoxville, Tennessee. Pediatric Exercise Science 2016 28(0) 35-36.

Young et al. The Internet is valid and reliable for child-report: an example using the Activities Scale for Kids (ASK) and the Pediatric Quality of Life Inventory (PedsQL). Journal of Clinical Epidemiology 2009 62(3) 314-20.

Zemel et al. Influence of complex childhood diseases on variation in growth and skeletal development. American Journal of Human Biology 2017 29(2).
